# Physicochemical properties and mechanism of action of a new copper(ii) pyrazine-based complex with high anticancer activity and selectivity towards cancer cells†

**DOI:** 10.1039/d4ra06874b

**Published:** 2024-11-12

**Authors:** B. Rogalewicz, T. Sierański, M. Szczesio, A. Olczak, K. Gobis, C. Orlewska, I. Korona-Głowniak, A. Korga-Plewko, M. Iwan, M. Michalczuk, J. Kubik, G. Adamczuk, M. Korga, N. Rutkowska, T. Boruta, K. Gas, M. Sawicki, E. Poleszak, W. Maniukiewicz, M. Świątkowski, A. Czylkowska

**Affiliations:** a Institute of General and Ecological Chemistry, Faculty of Chemistry, Lodz University of Technology Żeromskiego 116 Lodz 90-924 Poland bartlomiej.rogalewicz@dokt.p.lodz.pl agnieszka.czylkowska@p.lodz.pl; b Department of Organic Chemistry, Faculty of Pharmacy, Medical University of Gdansk Gen. Hallera 107 Gdańsk 80-416 Poland; c Department of Pharmaceutical Microbiology, Medical University of Lublin Lublin 20-093 Poland; d Independent Medical Biology Unit, Medical University of Lublin Jaczewskiego 8b Lublin 20-093 Poland; e Department of Toxicology, Medical University of Lublin Chodźki 8b Lublin 20-093 Poland; f Institute of Molecular and Industrial Biotechnology, Lodz University of Technology Stefanowskiego 2/22 Lodz 90-537 Poland; g Faculty of Process and Environmental Engineering, Department of Bioprocess Engineering, Lodz University of Technology ul. Wolczanska 213 Lodz 93-005 Poland; h Institute of Physics, Polish Academy of Sciences Aleja Lotnikow 32/46 Warsaw PL-02668 Poland; i Laboratory of Preclinical Testing, Chair and Department of Applied and Social Pharmacy, Medical University of Lublin Chodzki 1 Lublin 20-093 Poland

## Abstract

Two compounds, benzyl-2-(amino(pyrazin-2-yl)methylene)-1-methylhydrazine-1-carbodithioate (L) and its copper(ii) complex Cu(L) were synthesized and studied in terms of their physicochemical properties, including single crystal, spectroscopic and magnetic properties; *in silico* simulations, including DFT calculations and pharmacokinetic profile analysis; and *in vitro* biological activity. The Cu(L) compound was found to exhibit good anticancer activity against A375, PANC-1, MKN-74, T-47D, HeLa, and NCI-H1563 cells, with the IC_50_ value against the HeLa cell line reaching 17.50 μM, significantly surpassing the activity of the organic ligand. Moreover, at the same time, the Cu(L) complex did not exhibit significant toxicity towards healthy cells. Mechanism of action studies revealed that its activity is connected with the oxidative stress and redox imbalance caused by the upregulation of genes encoding superoxide dismutase (SOD2) and catalase (CAT) antioxidant enzymes. The reported results further underscore the anticancer potential of pyrazine-based copper(ii) complexes.

## Abstract 2

The presented work underlines the importance of coordination compounds in the development of new anticancer agents that are safer and more efficient.

## Graphical abstract

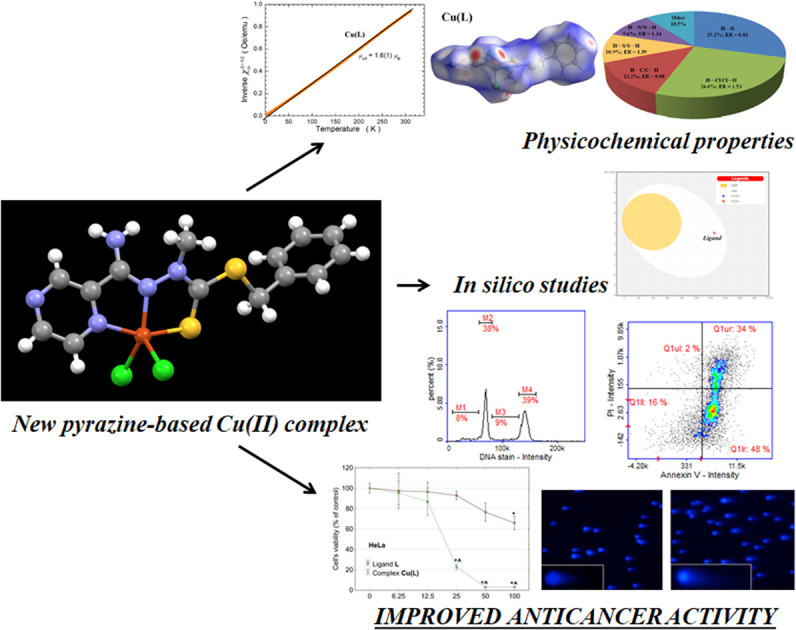

## 1. Introduction

With 24.6 million estimated new cases in 2030, cancer remains one of the deadliest diseases in the modern world.^1^ The most common therapeutic approaches include chemotherapy, radiation therapy, surgery, and hormonal therapy. Despite several disadvantages, chemotherapy remains at the forefront of the treatment, especially when alternative approaches such as radiation or surgery cannot be applied.^2,3^ With the growing resistance to some of the currently used drugs and collateral damage to healthy tissues, which result in severe adverse side effects, the search for new, more efficient, and safer therapeutics seems to be of superior importance. One of the most promising approaches is the utilization of coordination compounds containing metals lighter than platinum or ruthenium, which are mostly regarded as less toxic than heavier ones, which decreases the possibility of the occurrence of severe side effects. Current leading metals in this effort include copper and iron.^4,5^ One of the advantages of coordination chemistry, in terms of medicinal chemistry, is the possible synergistic effect of metal cations and organic ligands. This in turn allows for tailoring the properties of compounds and maximizing the possibility of obtaining compounds with good activity and low toxicity towards healthy cells. Currently, nitrogen- and sulfur-based compounds have attracted scientists' attention owing to their significant anticancer potential and remarkable coordinating properties.^6–8^

Copper complexes are known for their redox-active properties under physiological conditions, which have led to the synthesis and biological characterization of a number of copper-containing anticancer drugs and radiopharmaceuticals. Intracellular generation of reactive oxygen species (ROS) *via* thiol-mediated reduction of copper(ii) to copper(i) has been assumed as the major mechanism underlying the anticancer activity of copper(ii) complexes. Cancer cells have higher basal levels of ROS in comparison to normal cells. However, their redox homeostasis is maintained by the increased capacity of the antioxidant defense system, which involves several compounds and enzymes such as glutathione (GSH), peroxiredoxin (Prx), thioredoxin (Trx), superoxide dismutase (SOD) and catalase. Thus, an increase in ROS levels and/or inhibition of antioxidant processes causes redox imbalance and disrupts cancer cells homeostasis, which in turn leads to oxidative stress as well as lipid, protein and DNA damage, and ultimately cell death.^9–11^

NNS-coordinating ligands containing pyrazine or dithiocarbazate moieties have attracted ever-growing attention due to their excellent coordinating and biological properties. Their Cu(ii) complexes are known to exhibit significant anticancer activity.^12,13^ At the same time, only three structures containing both pyrazine and dithiocarbazate moieties were reported, although their biological potential was not investigated.^14^ NNS-coordinating Cu(ii) dithiocarbazate complexes bearing the pyridine moiety have been better characterized in the literature, with some of those complexes exhibiting strong activity against pancreatic, breast, and brain cancer cells.^15–17^

Considering the above rationale, we designed, synthesized, and studied the physicochemical properties and biological activity of a new copper(ii) pyrazine-based complex bearing both pyrazine and dithiocarbazate moieties, Cu(L). Analytical techniques used to study the complex included single crystal and powder X-ray diffraction analyses (SC-XRD and PXRD); elemental analysis (EA); inductively coupled plasma (ICP), infrared (FTIR), UV-visible (UV-vis) and fluorescence spectroscopies; and magnetic studies. The physicochemical analysis was supported with *in silico* methods, like Hirshfeld surface and fingerprint plots analyses, electrostatic potential maps and DFT calculations. *In silico* ADME analysis gave insight into the biological potential and pharmacokinetic profile of ligand L. In the last step, the *in vitro* activity against cancer and normal cells was studied, as well as on a panel of different bacterial strains and yeasts. For anticancer activity, mechanism of action was studied in detail, including apoptosis/necrosis detection, cell cycle analysis, thiol levels, ROS-related gene expression and DNA damage analysis.

## 2. Experimental

### 2.1 Chemistry and synthesis

All reagents used for the syntheses were purchased from Sigma-Aldrich and Avantor Performance Materials Poland companies, and used without further purification. For the reaction control, thin-layer chromatography was performed on Merck silica gel 60F_254_ plates and visualized with UV light. ^1^H and ^13^C NMR spectra were recorded on an INOVA-500 spectrometer (Fig. S1 and S2†). The melting point was determined on a Boetius apparatus (HMK, Dresden) and is uncorrected. The contents of C, H, N, and S were determined by a Vario MICRO company Elementar Analysensysteme GmbH. The content of Cu was determined by ICP spectroscopy. A standard solution from Merck (1000 mg L^−1^) was used for the preparation of the diluted solutions used for calibration. For analysis, distilled water (electrical conductivity = 0.05 μS cm^−1^) was used (obtained with the Polwater system). Solid samples were decomposed using the Anton Paar Multiwave 3000 closed-system instrument. Mineralization was carried out for 45 min at 240 °C under a pressure of 60 bar. Accurate mass measurements were obtained with the use of a high-resolution Q-TOF mass spectrometer SYNAPT G2 (Waters Corporation, Milford, MA, USA) under ESI+ mode (Fig. S3†). The following analytical conditions were applied: capillary 3.00 kV, sampling cone 25 V, extraction cone 4.0 V, source 120 °C, desolvation gas 500 L h^−1^. UV-vis spectra were acquired using a Jasco V-660 spectrophotometer, with solid samples compressed between two quartz plates. An integrating sphere was employed for this measurement. Solution measurements were performed for both compounds at a concentration of 5 μM in a DMSO/H_2_O (1 : 1) mixture in a time-dependent manner to investigate their stability after 24 and 48 h. These solutions were analyzed using quartz cuvettes with a 1 cm optical path length. Three-dimensional fluorescence spectra were collected on a Jasco FP-6300 spectrofluorometer (Jasco, Easton, MD, USA), positioning the solid samples at a 30° angle relative to the incident beam. Both monochromators were set with a data pitch and bandwidth of 1 nm. The FTIR spectra were recorded using an IR Tracer-100 Shimadzu Spectrometer (Shimadzu Corporation, Kyoto, Japan) (4000–600 cm^−1^) with an accuracy of recording of 1 cm^−1^, using KBr pellets. Spectra were processed and analyzed using Spectragryph software.^18^

#### 2.1.1 Synthesis of the ligand L

**Fig. 1:**
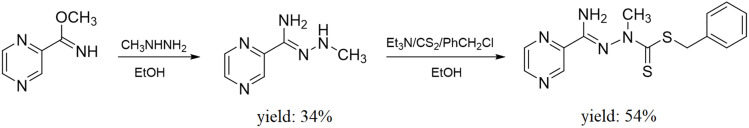
The synthesis route of the ligand L.

The starting material for the synthesis of the ligand L was methyl pyrazine-2-carbimidate, which was converted into *N*′-methylpyrazine-2-carbohydrazonamide in a reaction with methylhydrazine in ethanol, as reported previously.^19,20^ This intermediate was reacted with carbon disulfide and benzyl chloride in the presence of triethylamine in ethanol to obtain the final benzyl 2-(amino(pyrazin-2-yl)methylene)-1-methylhydrazine-1-carbodithioate (Fig. 1). *N*′-Methylpyrazine-2-carbohydrazonamide (0.520 g, 3.4 mmol) was suspended in methanol (10 mL), and trimethylamine (0.486 mL, 3.74 mmol) and carbon disulfide (0.211 mL, 3.74 mmol) were added. The mixture was stirred for 1 h at room temperature, and benzyl chloride was added (0.402 mL, 3.74 mmol). The mixture was stirred overnight and undissolved residues were filtered off. The filtrate was mixed with water and cooled. The precipitate was filtered off, washed with water, and recrystallized from methanol.^21^

Ligand L (C_14_H_15_N_5_S_2_) (benzyl-2-(amino(pyrazin-2-yl)methylene)-1-methylhydrazine-1-carbodithioate) (317.43 g mol^−1^) yield (54%) m.p. 140–141 °C. ^1^H NMR (500 MHz, DMSO-d_6_): *δ* 3.60 (s, 3H, NCH_3_), 4.36 (s, 2H, CH_2_), 7.20 (t, 1H, Ph, *J* = 7.33 Hz), 7.26 (t, 2H, Ph, *J* = 7.32 Hz), 7.34 (d, 2H, Ph, *J* = 7.32 Hz), 7.71 (br s, 2H, NH_2_), 8.73 (br s, 1H, pyrazine), 8.82 (d, 1H, pyrazine, *J* = 2.44 Hz), 9.25 (d, 1H, pyrazine, *J* = 0.97 Hz) ppm; ^13^C NMR (125 MHz, DMSO-d_6_): *δ* 42.29, 127.44, 128.80 (2C), 129.58 (2C), 137.81, 143.66, 144.01, 145.07, 147.39, 157.06, 191.37 ppm, signal of CH_3_ carbon overlapped with the solvent. Anal. calculated (%): C, 52.97; H, 4.76; N, 22.06. Found (%): C, 52.78; H, 4.64; N, 21.39. HRMS (ESI/Q-TOF) *m*/*z*: [M + H]^+^ calculated for C_14_H_16_N_5_S_2_: 318.0847; found: 318.0854 FTIR spectra (KBr, cm^−1^): *ν*(NH): 3410, 3309; *ν*(CH): 3024, 3000, 2970, 2931; *ν*(CN)_aliph_: 1647; *ν*(CC), *δ*(CH), *δ*(NH): 1580, 1561, 1452, 1416, 1367; *ν*(CN)_arom_: 1339; *ν*(NN): 1018; *ν*(CSS): 985.

#### 2.1.2 Synthesis of the complex Cu(L)

**Fig. 2:**
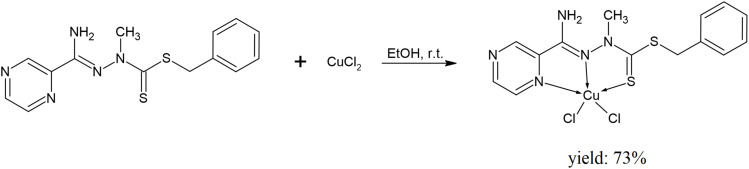
The synthesis route of the copper(ii) complex Cu(L).

The copper(ii) complex, Cu(L), was obtained in a single-step reaction between the ligand L and copper(ii) chloride dihydrate (Fig. 2). Equimolar amounts of the ligand and copper(ii) chloride dihydrate were dissolved in ethanol, and kept on a magnetic stirrer for 24 h at room temperature. After a couple of days, dark-green crystals of Cu(L) were filtered, washed a couple of times with diethyl ether, dried in open air, and analysed. Apart from SC-XRD measurements (for details, see Table S1†), PXRD measurements were also performed to confirm the sample's purity and homogeneity (Fig. S4†).

Complex Cu(L) (C_14_H_15_Cl_2_CuN_5_S_2_) (451.88 g mol^−1^) yield (73%) Anal. calculated (%): C, 37.21; H, 3.35; N, 15.50; S, 14.19; Cu, 14.06. Found (%): C, 37.11; H, 3.39; N, 15.28; S, 14.83; Cu, 14.51. HRMS (ESI/Q-TOF) *m*/*z*: [M − Cl]^+^ calculated for C_14_H_15_N_5_S_2_CuCl: 414.9753; found: 414.9760. FTIR spectra (KBr, cm^−1^): *ν*(NH): 3316; *ν*(CH): 2974, 2874; *ν*(CN)_aliph_: 1651; *ν*(CC), *δ*(CH), *δ*(NH): 1602, 1574, 1483, 1460, 1428, 1391; *ν*(CN)_arom_: 1364; *ν*(NN): 1038; *ν*(CSS): 1005.

### 2.2 SC-XRD analysis

Single crystals of the studied compound that were suitable for X-ray diffraction were obtained by slow solvent evaporation at room temperature from an ethanol solution. Diffraction measurements were performed using a XtaLAB Synergy, Dualflex diffractometer, (Rigaku Oxford Diffraction, Poland-Japan-UK), with a Pilatus 300 K detector at low temperature (100.0(2) K) using Mo Kα radiation (0.71073 Å). Diffraction data were processed using CrysAlisPRO software (Rigaku Oxford Diffraction. CrysAlisPRO; Rigaku Oxford Diffraction Ltd: Yarnton, Oxfordshire, England). Solving and refinement of the crystal structure were performed with SHELX^22,23^ using full-matrix least-squares minimization on F2. All H atoms were geometrically optimized and allowed as riding atoms, with C–H = 0.95 Å for aromatic CH groups, and 0.99 Å for secondary CH_2_ groups with Uiso(H) = 1.2 Ueq(C). In all studied structures, the methyl H atoms were refined with C–H = 0.98 Å and with Uiso(H) = 1.5 Ueq(C). ShelXle software^24^ was used to visualize the molecular structure. Graphical representation of the crystal structures was performed using the Mercury program.^25^ As a result of refinement of the crystal structure of the tested compound, a Hooft parameter equal to 0.5 was obtained. It turned out that this crystal is a merohedral twin, in which the two equivalent components are rotated 180° relative to each other around the OX or OY axis. Unfortunately, it is generally impossible to determine which rotation we are dealing with based on the diffraction data. The studied structure was refined with the matrix −1 0 0/0 −1 0/0 0 −1 because it is formally an inversion twin, but the same results can be obtained using the matrix 1 0 0/0 −1 0/0 0 −1 (rotation around the OX axis) or matrix −1 0 0/0 1 0/0 0 −1 (rotation around the OY axis). CCDC 2292685† contains the supplementary crystallographic data for this paper. These data can be obtained free of charge from The Cambridge Crystallographic Data Centre *via*www.ccdc.cam.ac.uk/structures. Hirshfeld surfaces and fingerprint plots were calculated and visualized using Crystal Explorer 17.5 (University of Western Australia, Nedlands 6009, Australia).^26^

### 2.3 PXRD analysis

Room temperature powder X-ray diffraction patterns of Cu(L) were collected using a PANalytical X'Pert Pro MPD diffractometer in the Bragg–Brentano reflection geometry (Cu K_α_ radiation, *λ* = 1.54059 Å, 40 kV/30 mA). The pattern was acquired in the 2*θ* range of 4–90° in a continuous scan mode with 0.0167° step size and an exposure per step of 30 s. The sample was rotated during the measurement to reduce the effect of preferred orientation in the crystalline phase.

### 2.4 Quantum-mechanical calculations

The excited states for the organic ligand L and its coordination compound Cu(L) were calculated using time-dependent density functional theory (TD-DFT) based on coordinates determined *via* X-ray measurements. Input structural models were prepared using Mercury 2022.3.0 software (Cambridge Crystallography Data Centre, Cambridge, UK).^25^ Hydrogen atom positions were normalized by adjusting them along the covalent bond vector (X → H) to a distance equivalent to the average neutron diffraction value. For the coordination compound, only the coordination unit was initially used as input. Calculations were carried out using Gaussian09 rev. E.01,^27^ employing the B3LYP density functional and 6-31g(2d,2p) basis set.^28–30^ To ensure the inclusion of all experimentally determined maxima, the number of calculated transitions was set to 500. Assignment of the calculated excited states to the observed experimental maxima was performed by comparing excitation energies and oscillator strengths/intensities of the corresponding maxima.

Furthermore, the Cu(L) complex was optimized and calculated in dimethyl sulfoxide (DMSO) as a solvent, utilizing the polarizable continuum model (PCM) to account for solvent effects.^31^ To assess the energetic stability of the compound in an aqueous environment, optimization of Cu(L) was also performed in water using the same solvent model. The computational methods, including the basis set, remained consistent with those used for the systems with X-ray derived coordinates.

The nature of the specific orbital excitations was analysed based on orbital contour plots. All orbital transitions were further examined using Chemissian v4.67 software.^32^ Molecular electrostatic potential maps (MESP) of the studied compounds were also generated, based on electron density calculations performed using the B3LYP/6-31g(2d, 2p) method.

### 2.5 Magnetic properties

5.6(2) and 2.1(2) mg sized samples of the ligand L and its coordination compound Cu(L), respectively, were magnetically investigated in a superconducting quantum interference device (SQUID) magnetometer MPMS XL of Quantum Design (San Diego, CA, USA) in magnetic field *H* up to several tenths of kOe and in temperature *T* down to 2 K. For the measurements, the powders were transferred into polycarbonate capsules (obtained also from Quantum Design). To facilitate accurate magnetic measurements of such miniscule powder specimens in much larger and heavier capsules (with a typical mass of about 33 mg), a recently described method of *in situ* compensation of the magnetic signal of the sample-carrying capsules was applied.^33^ By a careful selection of the abutting capsules, a compensation factor of above 40 has been obtained. During these measurements, the SQUID magnetometer was registering only 1/40 of the capsule carrying the powder specimen, thus reducing in the same proportion all uncertainties connected with determination of the searched signal of the powder, including very sizable errors.^33^ All the results analysed here are corrected for the (small) signals exerted by the empty sample holder, which are established separately. The measurements have been carried out following the code described for high-sensitivity studies of minute magnetic signals.^34^

### 2.6 *In silico* biological properties predictions

For *in silico* biological properties predictions, freely available online tools were used. The pharmacokinetic profile and druglikeness of the ligand L were analysed using the SwissADME service (Swiss Institute of Bioinformatics 2021).^35,36^ The overall toxicity was evaluated using ProTOX II service,^37^ and the cytotoxicity predictions towards cancer cells were calculated using CLC-Pred 2.0 (Cell Line Cytotoxicity Predictor).^38^

### 2.7 Anticancer activity

#### 2.7.1 Cell culturing

Cytotoxic activity of ligand L and its complex Cu(L) were evaluated against six human cancer cell lines: malignant melanoma A-375, pancreatic cancer PANC-1, gastric cancer MKN-74, breast cancer T-47D, cervical cancer HeLa and non-small cell lung cancer NCI-H1563. Additionally, the cytotoxicity of the tested compounds was evaluated against normal human fibroblast BJ. All cell lines originated from the American Type Culture Collection (ATCC). A375 and PANC-1 were cultured in Dulbecco's modified Eagle's medium (DMEM) (Corning, New York City). MKN-74 and T-47D were cultured in RPMI-1640 Medium (Corning, New York City). HeLa cells were cultured in Eagle's Minimum Essential Medium (EMEM) (Corning, New York City). All of these culture media were supplemented with 10% heat-inactivated fetal bovine serum (PAN-Biotech, Aidenbach, Germany), 100 μg per mL penicillin, 100 μg per mL streptomycin, and 0.25 μg per mL amphotericin B (Sigma-Aldrich, St. Louis, MO, USA). Cultures were maintained at 37 °C in a humidified atmosphere of 95% air and 5% CO_2_. The experiments were performed using cells from passages 5 to 10.

#### 2.7.2 MTT tests

The cells were plated in 96-well culture plates (2 × 10^4^ cells per well for all cell lines except the BJ fibroblast, which was seeded with 4 × 10^4^ cells per well density) and incubated in a CO_2_ incubator. The next day, treatment was given according to the experimental requirement. The cells were incubated with the tested compounds in concentrations ranging from 6.25 to 100 μM or DMSO as a vehicle in control cultures for 48 h (max. DMSO concentration <0.5%). The cytotoxicity of the tested compounds was evaluated using the MTT (methylthiazolyldiphenyl-tetrazolium bromide) test. MTT solution (4.0 mg mL^−1^) was added to the culture after 48 h of incubation. After 4 h, the medium with MTT was removed, and the formed crystals were dissolved in DMSO (200 μL per well). The solution absorbance was measured at 570 nm using the PowerWave microplate spectrophotometer (BioTek Instruments, USA). The experiment was performed twice with three replicates for each concentration of the tested compounds. IC_50_ values were determined using the AAT Bioquest IC_50_ calculator.^39^ Similarly, IC_50_ values were determined for copper(ii) chloride alone, as well as for standard chemotherapeutic agents: cisplatin for MKN-74, HeLa and NCI-H1563 cells, dacarbazine for A375, gemcitabine for PANC-1 and doxorubicin for T-47D cells.

#### 2.7.3 Statistical analysis

Statistical analysis and charts for the MTT test results were performed using STATISTICA 13 software (StatSoft, Krakow, Poland). Comparison of the values was performed by one-way analysis of variance (ANOVA) and post hoc multiple comparisons with Tukey's honest significant difference test (Tukey's HSD test). The data were calculated as mean ± SD. The differences were considered to be significantly different statistically if the *P* values were less than 0.05.

#### 2.7.4 Apoptosis detection

Apoptosis detection was performed using the NC 3000 system (ChemoMetec, Lillerød, Denmark) according to the manufacturer's protocol for the annexin V assay. The cells were seeded in 6-well culture plates at a density of 1 × 10^5^ cells per mL. After 70–80% confluence was achieved, they were treated with the complex Cu(L) in a concentration corresponding to the IC_50_ value obtained in the MTT test, and the ligand L in a concentration equal to 100 μM or DMSO as a vehicle in the control cultures, and analyzed after 48 h of incubation. The cells were dissociated into single-cell suspensions in PBS (Corning, New York, NY, USA). Approximately 4 × 10^5^ cells were resuspended in 100 μL of annexin V binding buffer, and incubated with annexin V-CF488A conjugate and Hoechst 33 342 for 15 min at 37 °C. Next, the cells were washed with annexin V binding buffer and the cell pellets were resuspended in 100 μL of annexin V binding buffer supplemented with 10 μg mL^−1^ of propidium iodide (PI), and the annexin V Assay was performed immediately. Cellular fluorescence was quantified using a NucleoCounter® NC-3000™ image cytometer (ChemoMetec USA Inc., Lillerød, Denmark).

#### 2.7.5 Cell cycle analysis

The cell cycle was analysed using the NC 3000 system (ChemoMetec, Lillerød, Denmark) according to the manufacturer's protocol for the 2-step cell cycle assay. The cells were seeded in 6-well culture plates at a density of 1 × 10^5^ cells per mL. After 70–80% confluence was achieved, they were treated with the complex Cu(L) in a concentration corresponding to the IC_50_ value obtained in the MTT test, and the ligand L in a concentration equal to 100 μM or DMSO as a vehicle in the control cultures, and analyzed after 48 h of incubation. The cells were washed with PBS (Corning, New York, NY, USA), and incubated with lysis buffer (solution 10) supplemented with 10 μg mL^−1^ of DAPI for 5 min at 37 °C. After addition of the stabilization buffer (solution 11), the samples were analysed. The fluorescence signal was quantified using a NucleoCounter® NC-3000™ image cytometer (ChemoMetec, Lillerød, Denmark).

#### 2.7.6 Analysis of intracellular thiol levels

To evaluate the intracellular thiol level, HeLa cells were seeded in a 6-well culture plate at a density of 2 × 10^5^ cells per mL and the tested compounds were added after reaching 70–80% confluence. After incubation for 48 h, the cells were washed with PBS and detached with trypsin–EDTA, followed by collection in a tube, centrifugation for 5 minutes, removal of the supernatant, and suspension of the cells with PBS. Subsequently, 10 μL of solution 5 (VB-48™·PI·AO) was added into 190 μL of the cell suspension. Next, the stained cells were loaded into 8-chamber NC-Slides A8™ and measured using the NC-3000 image cytometer (ChemoMetec, Lillerød, Denmark) according to the manufacturer's protocol for cell vitality assay. Solution 5 contains three different fluorescent dyes: acridine orange (AO) staining all cells, propidium iodide (PI) staining dead cells only, and VitaBright-48™ (VB-48™) which stains viable cells in an intensity that is dependent on their level of thiols. A high fluorescence intensity of a particular cell indicates that the cell has a high level of thiols such as GSH. The obtained VB-48™ fluorescence intensity scatter plots showing the distribution of cells based on their thiol levels were used to demarcate the healthy and low vitality cell subpopulations of the tested samples.

#### 2.7.7 Quantitative real-time PCR analysis (qRT-PCR)

Gene expression levels were determined by quantitative real-time PCR (qRT-PCR). HeLa cells were seeded into 25 cm^3^ flasks at a concentration of 2 × 10^5^ cells per mL, and test compounds were added after reaching 70–80% confluence. After 48 h of incubation, cells were treated with trypsin and harvested by centrifugation at 400 × *g* for 5 min at room temperature. Then, 1 mL of TRIzol™ Reagent (Invitrogen, Carlsbad, CA, USA) was added to lyse the cells. Lysates were centrifuged for 5 min at 12 000 × *g* at 4 °C, and clear supernatants were processed according to the manufacturer's protocol. For cDNA synthesis, all obtained samples (OD_260/280_ ratio of app. 2.0) were reverse-transcribed using NG dART RT-PCR reagents (EURx, Gdansk, Poland) and a Mastercycler gradient thermocycler (Eppendorf, Hamburg, Germany) according to the manufacturer's instructions, maintaining a reaction thermal profile of 10 min at 25 °C, 50 min at 50 °C and 5 min at 85 °C. The qPCR reaction was performed using Fast SG/ROX qPCR Master Mix reagents (2×) (EURx, Gdansk, Poland) in a 7500 fast real-time PCR system (Applied Biosystems, USA) in triplicate, according to the manufacturer's instructions. The thermal profile of the reactions was as follows: 20 s at 95 °C, followed by 40 cycles of 3 s at 95 °C and 30 s at 60 °C. The sequences of primers used in the qPCR are presented in Table S2.† RNA18SN5 and ACTB were used as reference genes, and the relative expression of the tested genes was determined by the ΔΔCt method. Analysis was performed using RQ values (relative quantification, RQ = 2^−ΔΔCt^). The data have been obtained from two independent experiments.

#### 2.7.8 Comet assay

The genotoxicity of the compounds was determined using the comet assay. Following 48 h of incubation with tested compounds as well as H_2_O_2_ (at 75 μM) as a positive control, the cells were detached from the 6-well plate using a trypsin–EDTA solution (Corning, New York, NY, USA), and they were thoroughly resuspended in DPBS (Corning, New York, NY, USA) lacking divalent cations. The final concentration of HeLa cells in each sample was adjusted to 1 × 10^5^ cells per mL. The comet assay was performed under neutral conditions (pH 8.5), as described previously by Olive *et al.*^40^ The cell suspensions (0.4 mL) were mixed with 1.2 mL of 1% (w/v) low melting point agarose (EURx, Gdansk, Poland), and then distributed onto microscope slides coated with 1% (w/v) normal melting agarose (EURx, Gdansk, Poland), covered with a cover slip, and kept for 10 minutes at 4 °C to solidify. Then, the microscope slides were submerged in a covered dish containing N1 lysis solution (2% sarkosyl, 0.5 M Na_2_EDTA, 0.5 mg mL^−1^ proteinase K (pH 8.0)) and kept at 37 °C overnight in the dark. After overnight lysis, the slides were submerged twice in room temperature N2 rinse buffer (90 mM Tris buffer, 90 mM boric acid, 2 mM Na_2_EDTA (pH 8.5)) for 30 minutes and placed horizontally in an electrophoresis tank side by side. Electrophoresis in solution N2 was run for 25 minutes at 0.6 V cm^−1^. After electrophoresis, the slides were washed for 15 minutes in distilled water, and stained using Hoechst 33342 (5 μg mL^−1^) for another 20 minutes. Comet images were captured using a Nikon Eclipse Ti inverted microscope with NIS-Elements Imaging Software (Nikon, Tokyo, Japan). Fifty individual cells were selected for calculations for each analysis. All experiments were performed at least three times, each with two parallel slides per data point. The images were analyzed using OpenComet (v1.3) a plugin for the image processing program ImageJ (Rasband, W.S., ImageJ, U. S. National Institutes of Health, Bethesda, Maryland, USA, https://imagej.net/ij/, 1997–2018.), which directly gives the % DNA in tail, tail length, and tail moment (TM). The parameter TM is the product of the tail length and % DNA in the tail.

### 2.8 Antimicrobial and antifungal activity

The minimal inhibitory concentration (MIC) and the minimum bactericidal/fungicidal concentration (MBC/MFC) of the tested complexes were evaluated for antibacterial and antifungal activity using the microdilution broth method in Mueller–Hinton broth or RPMI with MOPS for the growth of bacteria and fungi, respectively. The panel of reference microorganisms, including Gram-negative bacteria (*Escherichia coli* ATCC 25922, *Salmonella typhimurium* ATCC 14028, *Klebsiella pneumoniae* ATCC 13883, *Pseudomonas aeruginosa* ATCC 9027, *Proteus mirabilis* ATCC 12453), Gram-positive bacteria (*Staphylococcus aureus* ATCC 25923, *Staphylococcus aureus* ATCC BAA-1707, *Staphylococcus epidermidis* ATCC 12228, *Micrococcus luteus* ATCC 10240, *Enterococcus faecalis* ATCC 29212, *Bacillus cereus* ATCC 10876), and fungi (*Candida albicans* ATCC 10231, *Candida parapsilosis* ATCC 22019, *Candida glabrata* ATCC 90030) was investigated. The sterile 96-well polystyrene microtitration plates (Nunc, Denmark) were prepared by dispensing 100 μL of an appropriate dilution of the tested compounds in broth medium or per well by serial two-fold dilutions to obtain final concentrations of the tested compounds, ranging from 500 to 3.9 mg L^−1^. The inocula were prepared with fresh microbial cultures in sterile 0.85% NaCl to match the turbidity of 0.5. The McFarland standard was added to wells to obtain a final density of 5 × 10^5^ CFU mL^−1^ for bacteria and 5 × 10^4^ CFU mL^−1^ for yeasts (CFU, colony forming units). After incubation (35 °C for 24 h), the MICs were assessed visually and spectrophotometrically at 600 nm as the lowest concentration of the compounds that shows complete growth inhibition of the reference microbial strains. An appropriate DMSO control (at a final concentration of 10%), a strain growth control (inoculum without the tested compounds), and negative control (the tested compounds without inoculum) were included on each microplate. MBC or MFC was obtained by culture of 5 μL from each well that showed through growth inhibition, from the last positive one, and from the growth control onto recommended agar plates. The plates were incubated at 35 °C for 24 h for all microorganisms. The MBC/MFC was defined as the minimum concentration of compounds that kills 99.9% of the test microorganisms in the original inoculum. Vancomycin (Van), ciprofloxacin (Cip), and nystatin (Nys) were used as the standard reference antibiotics.

## 3. Results

### 3.1 SC-XRD analysis

**Fig. 3:**
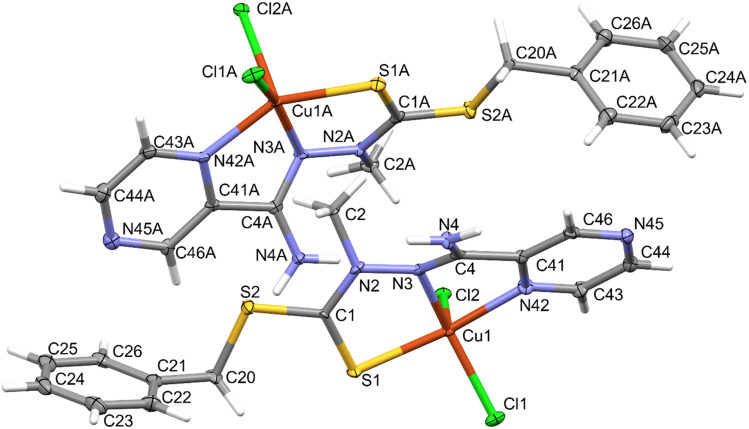
Molecular structure of the compound showing atom-labeling schemes. Displacement ellipsoids are drawn at the 50% probability level.

**Fig. 4:**
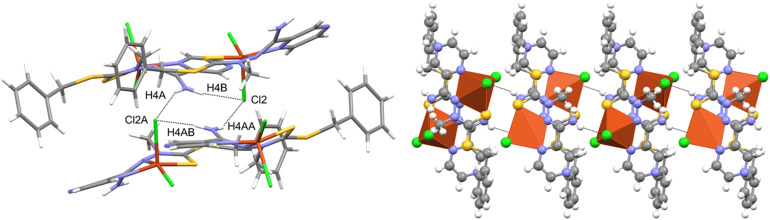
Intermolecular hydrogen bonds in the Cu(L) compound with atom labeling (left) and coordination polyhedra (right).

The compound crystallized in the space group *Pna*2_1_ with two molecules of the neutral complex in the asymmetric unit (Fig. 3). In both molecules, the copper atom is coordinated by two nitrogen atoms of the organic ligand, a thione sulfur atom, and two chlorine ions (Table 1). The neighborhood of the Cu^2+^ coordination atom may be described as distorted trigonal bipyramidal. Chlorine atoms and a hydrazine nitrogen atom occupy the equatorial positions. The structure is stabilized by hydrogen bonds of the N–H⋯Cl type (Fig. 4 and Table 2), which form the R2,4(8) ring according to the hydrogen-bond graph-set theory.^41^

**Table 1:** Geometric parameters (Å, °) for the Cu(L) compound

	Distance (Å)		Distance (Å)
Cu1A—Cl1A	2.2420 (15)	Cu1–Cl1	2.2587 (14)
Cu1A—Cl2A	2.4832 (14)	Cu1–Cl2	2.4789 (14)
Cu1A—S1A	2.3030 (15)	Cu1–S1	2.3060 (15)
Cu1A—N3A	1.976 (5)	Cu1–N3	1.991 (5)
Cu1A—N42A	2.062 (5)	Cu1–N42	2.050 (5)

	Angle (°)		Angle (°)
Cl1A—Cu1A—Cl2A	100.18 (5)	Cl1–Cu1–Cl2	99.83 (5)
Cl1A—Cu1A—S1A	95.33 (6)	Cl1–Cu1–S1	95.06 (6)
S1A—Cu1A—Cl2A	102.28 (5)	S1–Cu1–Cl2	102.29 (5)
N3A—Cu1A—Cl1A	157.38 (14)	N3–Cu1–Cl1	146.63 (14)
N3A—Cu1A—Cl2A	102.18 (14)	N3–Cu1–Cl2	113.14 (14)
N3A—Cu1A—S1A	83.22 (16)	N3–Cu1–S1	83.21 (16)
N3A—Cu1A—N42A	78.2 (2)	N3–Cu1–N42	77.2 (2)
N42A—Cu1A—Cl1A	93.96 (14)	N42–Cu1–Cl1	93.80 (15)
N42A—Cu1A—Cl2A	102.01 (14)	N42–Cu1–Cl2	98.08 (14)
N42A—Cu1A—S1A	151.97 (14)	N42–Cu1–S1	156.01 (14)

**Table 2:** Hydrogen-bond geometry (Å, °) for the Cu(L) complex^a^

D—H⋯A	D—H	H⋯A	D⋯A	D—H⋯A
N4A—H4AA⋯Cl2	0.88	2.50	3.300 (5)	152
N4A—H4AB⋯Cl2A^i^	0.88	2.37	3.199 (5)	158
N4–H4A⋯Cl2A^ii^	0.88	2.48	3.289 (5)	154
N4–H4B⋯Cl2^iii^	0.88	2.45	3.275 (5)	156

a Symmetry codes: (i) *x* + 1/2, −*y* + 5/2, *z*; (ii) *x*, *y* − 1, *z*; (iii) *x* − 1/2, −*y* + 3/2, *z*.

### 3.2 Hirshfeld surface analysis and electrostatic potential maps

Hirshfeld surface analysis is a useful tool for evaluating the intermolecular interactions with the neighboring molecules within crystals. For both compounds, L and Cu(L), Hirshfeld surfaces and the fingerprint plots were generated and studied (Fig. S5–S7†). For all described contacts, enrichment ratio (ER) values were calculated to determine whether or not a contact is favored in a crystalline environment.^42^ For the L compound, four main contacts were detected: H⋯H (44.6%), H⋯C/C⋯H (17.5%), H⋯S/S⋯H (17.2%), and H⋯N/N⋯H (12.2%). With ER = 1.42, the H⋯S/S⋯H contacts seem to play a key role in the crystal structure of L, as these contacts are the most favored ones. The ER values for the remaining contacts are close to 1. The key difference in the Cu(L) contacts is the crucial role of the H⋯Cl/Cl⋯H contacts in the structural assembly, both in terms of the percentage share (26.6%) and high ER value (1.53). The distribution of the remaining contacts is similar, with H⋯H (29.2%) and H⋯C/C⋯H (13.2%), and the highly favored H⋯S/S⋯H (10.9%) and H⋯N/N⋯H (9.6%) contacts.

**Fig. 5:**
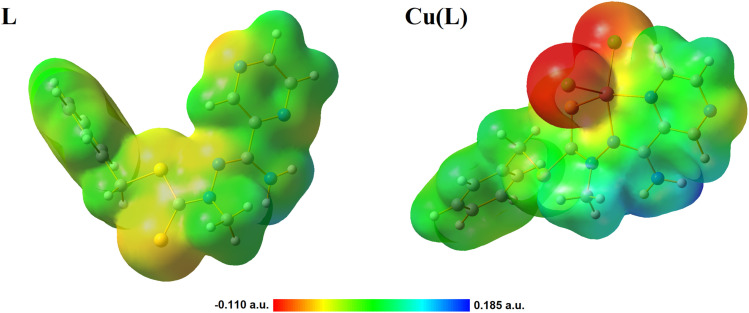
Molecular electrostatic potential maps (MESP) of the studied compounds imposed on electron density at an isovalue of 0.005.

For both compounds, electrostatic potential maps (MEPS) were generated and analyzed (Fig. 5). MEPS offer a three-dimensional illustration of a molecule's charge distribution. For the organic ligand alone, a negative charge is predominantly localized on the sulfur atoms. However, in the coordination compound containing chlorine atoms, there is a noticeable shift, with the negative charge becoming more pronounced and concentrated on the chlorine atoms. The coordination induces a change in the molecule's geometry and, consequently, its charge distribution. In both scenarios, the NH_2_ group emerges as the most positively charged region. This pronounced positive charge is due to its electron deficiency, which is far more evident in the coordination compound.

### 3.3 Quantum-mechanical calculations, UV-vis, fluorescence and FTIR measurements

#### 3.3.1 Quantum-mechanical calculations, UV-vis and fluorescence spectra

For the solid state measurements, a notable correspondence between the TD-DFT calculated and experimental spectra is evident (Fig. S8†), elucidating the involved electronic transitions. For the organic ligand L, two main absorption maxima are identified. The first maximum is attributed to π → π* and *n*/π → π* transitions (Table S3†), predominantly involving the lone pair electrons on the sulfur atom (Fig. S9†). The second absorption maximum is also identified as an *n*/π → π* transition, in which the lone pairs of the sulfur atom are similarly engaged. In the case of the Cu(L) coordination compound, a more complex spectroscopic profile is revealed (Table S3†). The first maximum is linked to a blend of *n*/π → π* and more intricate orbital transitions, involving the electron density concentrated around the copper atom, which includes d-electron transitions. Subsequent maxima are ascribed to transitions where the acceptor orbital encompasses electron density focused around the copper atom's d-orbitals, sigma orbitals, and lone pairs of chloride ions (Fig. S9†). The donor orbital is found to include anti-bonding π and d-orbitals of the copper atom. Lastly, an absorption maximum appearing at higher wavelengths (above 700 nm) is attributed to *n*/d → π*/d transitions, implicating the lone pairs of the chloride ions and the d-orbitals of the copper atom (Table S3†).

In addition to solid-state measurements, the UV-vis spectrum for Cu(L) was also recorded in DMSO. Five distinct maxima were observed (Fig. S11 and Table S3†), closely corresponding to the calculated values. More pronounced differences are visible for transitions at high wavenumbers (Fig. S11 and Table S3†), which are typical for TD-DFT calculations in this range. This is related to the fact that the observed maximum in the spectrum is actually associated with several different energy transitions. The nature of the transitions is similar to those observed in the solid state and mainly concerns transitions in which the acceptor orbital is an anti-bonding π orbital (Fig. S11†). However, in aqueous solution, the influence of the d orbitals of the central atom (copper) becomes more apparent. It can be observed that in most cases, the electron density of the donor orbital is also localized around the copper atom. All described transitions can therefore be classified as *n* → π* and d → π* transitions, except for the transition associated with the first maximum, where the acceptor orbital is the anti-bonding σ orbitals of the organic ligand. In general, the transitions have the character of a metal-to-ligand charge transfer (MLCT). Additionally, optimization of the Cu(L) complex was performed in a water environment. A decrease in energy was observed during the optimization process, and no dissociation of the complex occurred (Fig. S12†), suggesting that the obtained coordination compound remains intact in aqueous solutions. To assess the stability of both ligand L and its complex, Cu(L) in mixed DMSO/H_2_O solutions, their absorption spectra in DMSO/H_2_O (1 : 1) mixture were recorded in a time-dependent manner (Fig. S13†). No significant changes were observed after 24 and 48 hours, confirming the high stability of both compounds.

The organic ligand L also exhibits weak fluorescence with a single pronounced maximum, observed at an excitation wavelength of 470 nm and an emission wavelength of 481 nm (Table S3 and Fig. S14†). In contrast, the coordination compound Cu(L) essentially shows no fluorescence. This quenching effect is attributed to the copper cation, which has an open-shell electronic structure, and aligns with the general findings in the literature.^43^ Fluorescence quenching may be useful in applications such as biosensors, where specific quenching could serve as an indicator of the analyte presence.

#### 3.3.2 FTIR spectra

In the ligand L spectrum (Fig. S15†), sharp bands at 3410 and 3309 cm^−1^ can be attributed to the *ν*(NH) modes. Interestingly, in the spectrum of a Cu(L) complex (Fig. S16†), only one weak band at 3316 cm^−1^ can be observed. This can be explained by the strong hydrogen bonds formed between the chlorine atoms and amino group hydrogens, which correlates well with the crystallographic analysis results. In both spectra, several *ν*(CH) bands can be observed around 3000 cm^−1^. For both compounds, *ν*(CN), *ν*(CC), *δ*(CH), *δ*(NH), *ν*(NN), and *ν*(CS) bands can be found in the range between 1660 and 980 cm^−1^. Based on the literature, the Cu(L) NNS coordination mode, and the shift of some of these bands in comparison with the L spectrum, sharp bands at 1647 cm^−1^ for L and 1651 cm^−1^ for Cu(L) can be ascribed to the *ν*(CN)_aliph_ azomethine modes.^44^ Furthermore, the single bands at 1018 cm^−1^ can be ascribed to L, and those at 1038 cm^−1^ for Cu(L) are associated with the *ν*(NN) modes.^45^ As a result of coordination, both *ν*(CN)_arom_ pyrazine and *ν*(CSS) bands are shifted significantly: from 1339 cm^−1^ in L, to 1364 cm^−1^ in Cu(L), and from 985 cm^−1^ in L, to 1005 cm^−1^ in Cu(L), accordingly.^45^

### 3.4 Magnetic properties

In the first step, the molar magnetic susceptibility *χ*_m_ of the ligand L was established. The slope of its linear response in *H* at *T* = 300 K yields *χ*_m_^L^ = −1.63 × 10^−4^ emu mol^−1^. Under the same experimental conditions, the coordination compound Cu(L) exhibits a net paramagnetic response, which grows steadily on lowering T. This behavior can be assigned to the presence of spin *S* = ½ Cu ions with configuration 3*d*^9^ in the investigated compound. To evaluate the molar spin susceptibility of these ions, *χ*_m_^*S* = ½^, we corrected the experimental data for the magnitudes of *χ*_m_^L^ (noting that the L ligand constitutes only 70% of the mass of Cu(L) compound) and diamagnetic corrections for (i) the Cu^2+^ cation, *χ*_m_^Cu^ = −1.1 × 10^−5^ emu mol^−1^ and two Cl^−^ anions, *χ*_m_^Cl^−^^ = −2.34 × 10^−5^ emu mol^−1^.^46^ The resulting temperature dependence of the inverse of *χ*_m_^*S* = ½^ is presented in Fig. S17.† Indeed, the experimental points follow the paramagnetic *T*^−1^ dependency very closely, as indicated by the solid straight line. The slope of this line yields the magnitude of the inverse of the Curie constant *C*_mol^−1^_ = 3 *k*_B_/*μ*_eff_^2^/*N*_A_, from which the magnitude of the effective spin moment *μ*_eff_^2^ ≅ 8/*C*_mol_ of the Cu ions is readily obtained. Here, *μ*_eff_ is given in units of Bohr magnetons *μ*_B_, *k*_B_ is the Boltzmann constant, and *N*_A_ is the Avogadro number. We obtained *μ*_eff_^Cu^ = 1.6(1) *μ*_B_, which correlates remarkably well with the spin-only magnitude of 3*d*^9^ configuration, for which *μ*_eff_ = 1.73 *μ*_B_ is expected. This finding confirms the presence of a single unpaired electron on the Cu site and its 3*d*^9^ configuration. The uncertainty of *μ*_eff_^Cu^ is determined predominantly by the uncertainty of the masses of the powder specimens after transferring into the capsules.

### 3.5 *In silico* biological properties predictions

**Fig. 6:**
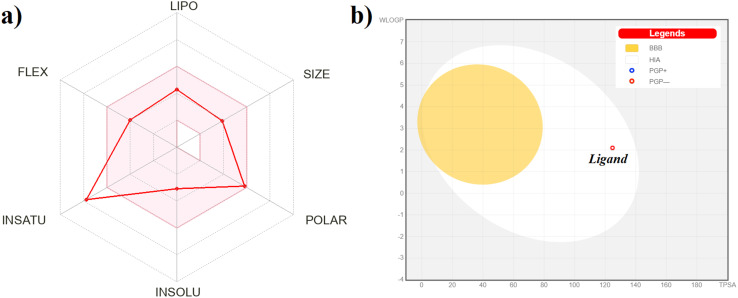
(a) Bioavailability graph generated using the SwissADME service. The red-colored zone is considered physicochemically suitable for oral bioavailability. LIPO, lipophilicity (−0.7 < XlogP3 < +5.0); SIZE, molecular weight (150 g mol^−1^ < MW < 500 g mol^−1^); POLAR, polarity (20 Å^2^ < TPSA < 130 Å^2^); INSOLU, insolubility (0 < log *S* < 6); INSATU – insaturation (0.25 < fraction Csp3 < 1); FLEX – flexibility (0 < num. of rotatable bonds < 9). (b) The BOILED-egg diagram for the compound L. The white area represents high gastrointestinal absorption; the yellow area represents blood–brain barrier permeation.

*In silico* tests covered the ADME bioavailability analysis and predictions of overall toxicity, as well as cytotoxicity towards cancer cells of the ligand L. Its pharmacokinetic profile was evaluated based on six basic properties (lipophilicity, size, polarity, solubility, flexibility, and saturation; Fig. 6a), and allowed to classify the ligand L as a potential drug candidate. It meets the rules of Lipinski,^47^ Ghose,^48^ Egan,^49^ Veber,^50^ and Muegge^51^ for druglikeness. The BOILED-egg diagram analysis suggests moderate solubility in water with high gastrointestinal absorption and no blood–brain-barrier permeation. Thus, compound L is predicted not to be the *P*-glycoprotein substrate (Fig. 6b). Therefore, it may be a suitable drug candidate with oral administration and a poor drug candidate against brain tumors. According to the overall toxicity predictions, compound L belongs to the 4th toxicity class. The predicted LD_50_ value was equal to 400 mg kg^−1^. The evaluation of the cytotoxicity towards cancer cell lines revealed good results for the tested compound, with the highest probability of anticancer activity against skin, cervix, and lung cancer cells (Fig. S18†). Moderate activity was predicted against pancreas, breast, stomach, prostate, and urinary bladder cancers, suggesting the above-mentioned diseases as potential targets.

### 3.6 Anticancer activity and mechanism of action

The *in vitro* cytotoxicity of both ligand L and complex Cu(L) was investigated on cell lines of different origin, such as A-375 (malignant melanoma), PANC-1 (pancreatic cancer), MKN-74 (gastric cancer), T-47D (breast cancer), HeLa (cervical cancer) and NCI-H1563 (non-small cell lung cancer), with BJ (normal human fibroblast) as the non-cancer control cell line. Results were compared with those of the widely used drugs (dacarbazine, gemcitabine, doxorubicin and cisplatin), as well as copper(ii) chloride alone, under the same conditions.

Cells were exposed to 0–100 μM of L or Cu(L) for 48 h, and cell viability was assessed using the MTT assay. Results were determined according to the dose values of the potential drug needed to reduce the cell lines growth to 50% (IC_50_). In the range of the concentrations used, the ligand L was slightly cytotoxic against the tested cell lines. The IC_50_ value was calculated only in the case of the MKN-74 gastric cancer line (Table 3 and Fig. S19†). Complexation of L with copper(ii) ions substantially increased the cytotoxic activity against cancer cells, without toxicity towards normal fibroblasts. Significant differences were observed starting from the concentration of 25 μM in the case of the HeLa cell line, and 50 μM for the other cell lines (Fig. S19†). Similarly, for the HeLa line, the lowest IC_50_ value was obtained. For A375 and HeLa cell lines, the cytotoxicity of Cu(L) was comparable to the cytotoxicity of the reference drugs, dacarbazine and cisplatin, respectively. At the same time, copper(ii) chloride alone did not exhibit significant toxicity in the tested concentrations.

**Table 3:** IC_50_ values based on the MTT test after 48 h incubation with ligand L and its complex Cu(L)

	A375	PANC-1	MKN-74	T-47D	HeLa	NCI-H1563	BJ
	IC_50_ (μM)						
Ligand L	>100	>100	89.2 ± 4.47	>100	>100	>100	>100
Complex Cu(L)	40.81 ± 1.18	46.90 ± 3.64	38.1 ± 0.78	44.71 ± 4.75	17.50 ± 2.44	39.11 ± 3.99	>100
Reference	47.12 ± 2.78 (dacarbazine)	12.05 ± 1.12 (gemcitabine)	15.02 ± 0.98 (cisplatin)	5.28 ± 0.32 (doxorubicin)	13.68 ± 1.21 (cisplatin)	2.25 ± 0.30 (cisplatin)	—
CuCl_2_	>100	>100	>100	>100	>100	>100	>100

**Fig. 7:**
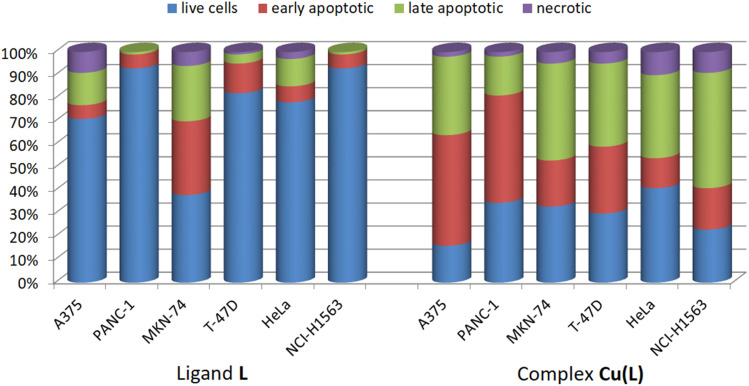
Cancer cell populations based on apoptosis/necrosis detection *via* annexin V and image cytometry.

In addition to the MTT assay, the cytotoxic activities of ligand L and its complex, Cu(L), were verified by apoptosis detection using annexin V and image cytometry. Annexin V binds specifically to phosphatidylserine, which is externalized on the outer surface of the cell membrane to mark the cell to undergo apoptosis process.^52^ Analysis of the ligand L activity confirmed its low cytotoxicity against the majority of the cell lines – only in the case of MKN-74, ligand L induced apoptosis at a concentration equal to 100 μM (Fig. 7 and S20†). The Cu(L) complex induced apoptosis, with a predominance of cells in the early apoptosis stage in the A375 and PANC-1 lines and late apoptosis predominance in the MKN-74, T46D, HeLa, and NCI-H1563 cell cultures (Fig. 7 and S20†).

**Fig. 8:**
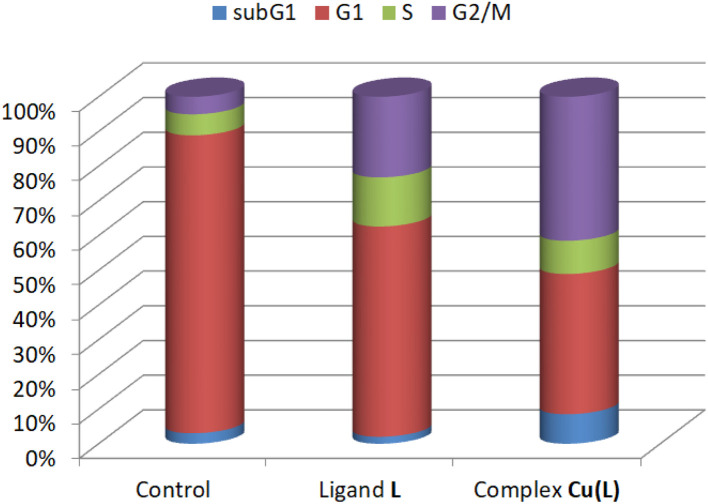
Cell cycle distribution, showing the populations of HeLa cells in different phases (subG1, G1, S, and G2/M) after 48 h of incubation with ligand L (100 μM), complex Cu(L) (17.50 μM), and a positive control.

As the lowest IC_50_ value was obtained for the complex Cu(L) against the HeLa cell line, it was subjected to cell cycle analysis. The concentration of Cu(L) corresponded to the IC_50_ value obtained in the MTT test, with the ligand L equal to 100 μM or DMSO used as a vehicle in the control cultures, and the culture was analyzed after 48 hours of incubation. Cell cycle analysis showed that treatment of HeLa cells with ligand L and complex Cu(L) resulted in an increase in the population of cells in the G2/M phase at the expense of the population in the S phase (Fig. 8 and S21†). This pattern was much more pronounced for Cu(L). Cell cycle arrest in the G2 phase indicates that the cell has halted its progression through the cell cycle due to various reasons, such as DNA damage, allowing the cell to undergo the DNA repair pathway.

Copper(ii) complexes are known to induce oxidative stress in cancer cells *via* redox cycling, which leads to the production of superoxide radicals (˙O^2−^) and hydrogen peroxide (H_2_O_2_).^12,53^ Cellular thiols, particularly glutathione (GSH), play a crucial role in protecting cells from oxidative stress.^11^ The cycle of oxidation and regeneration of glutathione (GSH) involves GSH donating electrons to neutralize reactive oxygen species (ROS), becoming oxidized (GSSG), and then being reduced back to its active form, GSH. A decrease in the level of reduced thiols shifts the cellular redox potential towards a more oxidative state, which can trigger oxidative damage to lipids, proteins, and DNA.^11^ Therefore, the level of cellular thiols was examined in HeLa cells, which showed the greatest sensitivity to the tested compounds.

**Fig. 9:**
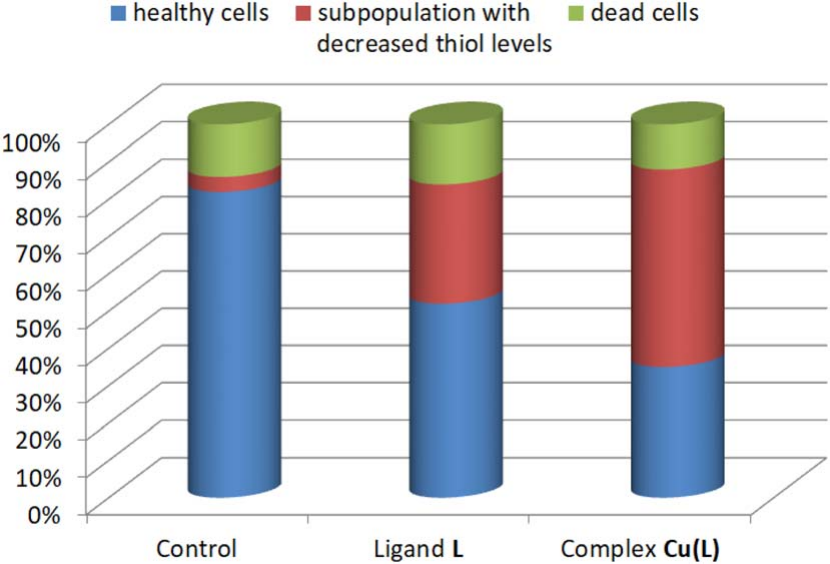
Thiol level distribution in HeLa cells after 48 h of incubation with the ligand L (100 μM), complex Cu(L) (17.50 μM), and positive control.

Analysis of the thiol level distribution by image cytometry revealed the presence of a subpopulation of cells with decreased thiol levels after treatment with ligand L and its complex Cu(L), equal to 32% and 53%, respectively, *vs.* 4% in the control culture (Fig. 9 and S22†).

**Fig. 10:**
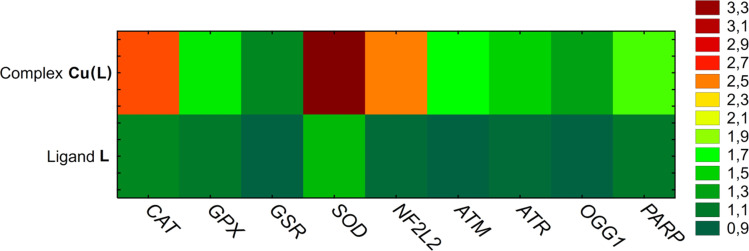
The results of mRNA gene expression analysis in HeLa cells treated with ligand L (100 μM) and complex Cu(L) (at concentrations corresponding to an IC_50_ value of 17.50 μM). Mean RQ levels are marked with a color scale.

Reduced thiol levels found in the studied cells indicate a redox imbalance. Thus, it is possible that the cycle arrest is related to oxidative stress and oxidative DNA damage. The expression of enzymes related to the first line of cell defense against reactive oxygen species (ROS) and DNA damage was evaluated. It was observed that Cu(L) complex treatment significantly upregulated expression of the *NF2L2* gene, which is a transcription factor for genes encoding antioxidant enzymes (such as superoxide dismutase (SOD2) and catalase (CAT)) neutralizing superoxide radicals and hydrogen peroxide, respectively. These two genes exhibited an approximately a 3-fold increase in the expression level. The expression of enzymes involved in the GSH oxidation and regeneration cycle (GPX and GSR), as well as the genes associated with oxidative DNA damage (ATM, ATR, OGG1, PARP), increased to a lesser extent. For comparison, the changes in the tested gene expression in HeLa cells after incubation with the ligand L were minor, with only SOD expression being increased by almost 1.5-fold (Fig. 10).

**Fig. 11:**
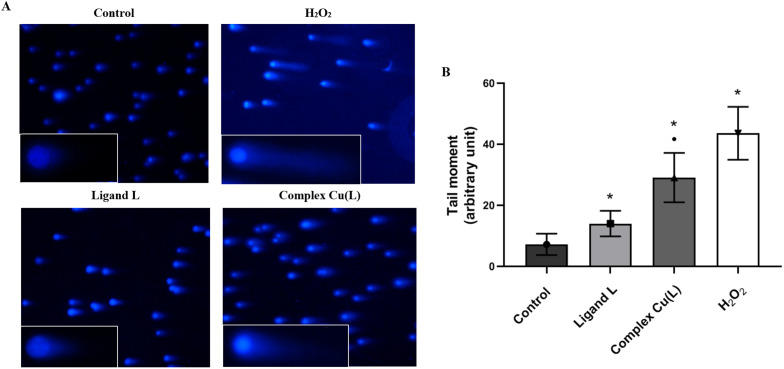
DNA damage (tail moment) induced by ligand L (100 μM) and complex Cu(L) (at concentration corresponding to an IC_50_ value of 17.50 μM) detected by the neutral comet assay in HeLa cells. (A) Representative images from the neutral comet assay (100× and 150× magnification). (B) Representative graph obtained by plotting the means of the tail moment (whose magnitude reflects the frequency of DNA strand breaks per nucleus) of the HeLa cells comets. The values are presented as the mean of three independent experiments ± SD. *p < 0.05 *vs.* control. ■ *p* < 0.05 *vs.* ligand L.

To determine the genotoxic effect of ligand L and its copper complex Cu(L), the comet assay was used, presented as the tail moment parameter (tail length × DNA amount in the tail). The tail moment values of the negative control (the untreated HeLa cells) indicated a low level of spontaneous DNA damage (TM: 7.22 ± 3.25) (Fig. 11). A higher degree of DNA degradation than that observed in the control was noted in cells treated with ligand L (14.01 ± 4.18) and the studied complex Cu(L) (29.1 ± 8.08), indicating both their genotoxic and cytotoxic properties. In addition, complex Cu(L) induces twice as much DNA damage as compared to ligand L.

### 3.7 Antimicrobial and antifungal activity

Antimicrobial activities of the free ligand L and its copper(ii) complex Cu(L) were tested against a wide panel of Gram-negative bacteria, Gram-positive bacteria, and yeasts (Table S4†). Whereas the ligand L showed a lack of activity in the tested range of concentrations, Cu(L) revealed negligible activity against the tested Gram-positive bacteria (MIC 250 mg L^−1^). Interestingly, comparable activity was observed against methicillin-resistant *Staphylococcus aureus* (MRSA). It was found that the Cu(L) complex exhibited bactericidal activity since the MBC/MIC index for most of the tested Gram-positives was <4. Cu(L) was also slightly active against two out of the three tested yeasts. Activity against Gram-negative bacteria was not observed for ligand L or for the Cu(L) complex. Vancomycin (Van), ciprofloxacin (Cip), and nystatin (Nys) were used as the standard reference antibiotics.

## 4. Conclusions

In the presented study, a new copper(ii) pyrazine-based complex bearing a dithiocarbazate moiety Cu(L) was synthesized and studied in terms of its physicochemical properties, *in silico* simulations, and biological activity. The NNS-coordinated Cu(L) complex is stabilized by hydrogen bonds formed between NH_2_ hydrogen atoms and chlorine atoms, as highlighted through SC-XRD, Hirshfeld surface and fingerprint plot analysis. Both the Cu(L) complex and its corresponding ligand L were tested for anticancer activity against A375, PANC-1, MKN-74, T-47D, HeLa, and NCI-H1563 cancer cells, and normal human fibroblasts BJ. Along with the increasing concentration of Cu(L), a significant increase in activity against all cancer cells was observed in comparison with the ligand L alone. The lowest IC_50_ value (17.50 μM) was reached against HeLa lines. For this cell line, the studies of the mechanism of action revealed that the high activity of this compound is related to the redox imbalance and oxidative stress induced in the treated cells, caused by the upregulation of genes encoding superoxide dismutase (SOD2) and catalase (CAT) antioxidant enzymes. Structures of a similar Cu(ii) NNS-coordinated dithiocarbazate complex bearing pyrazine moiety were reported, although their biological activity has not been studied.^14^ In contrast, complexes containing pyridine moieties have been more thoroughly investigated, often reaching sub-micromolar or near-sub-micromolar IC_50_ values.^15,16^ While ligand L was found to be mostly inactive, Cu(L) exhibited good anticancer activity. This trend can also be observed in the abovementioned pyridine-based complexes, highlighting the potential of the novel metalorganic drugs concept, especially in terms of the application of metals lighter than platinum or ruthenium (like copper), which are generally considered significantly less toxic. While the synthesized Cu(L) complex exhibited good anticancer activity, its antibacterial activity was found to be negligible.

## Data availability

Crystallographic data for Cu(L) has been deposited at the CCDC as CCDC 2292685.†

## Conflicts of interest

The Authors declare no conflict of interest.

## Supplementary Material

RA-014-D4RA06874B-s001

RA-014-D4RA06874B-s002

## References

[cit1] Cancer Tomorrow , https://gco.iarc.fr/tomorrow/en/dataviz/isotype?years=2040&single_unit=500000&sexes=0, accessed 24 August 2023

[cit2] Anand U., Dey A., Chandel A. K. S., Sanyal R., Mishra A., Pandey D. K., De Falco V., Upadhyay A., Kandimalla R., Chaudhary A., Dhanjal J. K., Dewanjee S., Vallamkondu J., Pérez de la Lastra J. M. (2023). Genes Dis..

[cit3] Tilsed C. M., Fisher S. A., Nowak A. K., Lake R. A., Lesterhuis W. J. (2022). Front. Oncol..

[cit4] Wani W. A., Baig U., Shreaz S., Shiekh R. A., Iqbal P. F., Jameel E., Ahmad A., Mohd-Setapar S. H., Mushtaque M., Hun L. T. (2016). New J. Chem..

[cit5] Ndagi U., Mhlongo N., Soliman M. (2017). Drug Des. Dev. Ther..

[cit6] Paprocka R., Wiese-Szadkowska M., Kosmalski T., Frisch D., Ratajczak M., Modzelewska-Banachiewicz B., Studzińska R. (2022). Pharmacy.

[cit7] Shakya B., Yadav P. N. (2020). Mini Rev. Med. Chem..

[cit8] Lang D. K., Kaur R., Arora R., Saini B., Arora S. (2020). Anticancer Agents Med. Chem..

[cit9] Kim S. J., Kim H. S., Seo Y. R. (2019). Oxid. Med. Cell. Longev..

[cit10] Marioli-Sapsakou G.-K., Kourti M. (2021). Anticancer Res..

[cit11] Baba S. P., Bhatnagar A. (2018). Curr. Opin. Toxicol..

[cit12] Santini C., Pellei M., Gandin V., Porchia M., Tisato F., Marzano C. (2014). Chem. Rev..

[cit13] Singh N. K., Kumbhar A. A., Pokharel Y. R., Yadav P. N. (2020). J. Inorg. Biochem..

[cit14] Hamid M. H. S. A., Akbar Ali M., Mirza A. H., Bernhardt P. V., Moubaraki B., Murray K. S. (2009). Inorg. Chim. Acta.

[cit15] Gou Y., Chen M., Li S., Deng J., Li J., Fang G., Yang F., Huang G. (2021). J. Med. Chem..

[cit16] Cavalcante C. D. Q. O., Garcia E., da Mota T. H. A., de Oliveira D. M., Gatto C. C. (2022). J. Inorg. Biochem..

[cit17] Santra A., Brandao P., Mondal G., Bera P., Jana A., Bhattacharyya I., Pramanik C., Bera P. (2020). Polyhedron.

[cit18] Menges F. , ‘Spectragryph – Optical Spectroscopy Software’, Version 1.2.16.1, 2022, http://www.effemm2.de/spectragryph/

[cit19] Czylkowska A., Rogalewicz B., Szczesio M., Raducka A., Gobis K., Szymański P., Czarnecka K., Camargo B. C., Szczytko J., Babich A., Dubkov S., Lazarenko P. (2022). Int. J.
Mol. Sci..

[cit20] Foks H., Balewski L., Gobis K., Dabrowska-Szponar M., Wisniewska K. (2012). Heteroat. Chem..

[cit21] Olczak A., Szczesio M., Gołka J., Orlewska C., Gobis K., Foks H., Główka M. L. (2011). Acta Crystallogr., C.

[cit22] Sheldrick G. M. (2015). Acta Crystallogr., A.

[cit23] Sheldrick G. M. (2008). Acta Crystallogr., A.

[cit24] Hübschle C. B., Sheldrick G. M., Dittrich B. (2011). J. Appl. Crystallogr..

[cit25] Macrae C. F., Sovago I., Cottrell S. J., Galek P. T. A., McCabe P., Pidcock E., Platings M., Shields G. P., Stevens J. S., Towler M., Wood P. A. (2020). J. Appl. Crystallogr..

[cit26] Spackman P. R., Turner M. J., McKinnon J. J., Wolff S. K., Grimwood D. J., Jayatilaka D., Spackman M. A. (2021). J. Appl. Crystallogr..

[cit27] Frisch M. J. , et al., Gaussian 09 (Revision E.01), 2013

[cit28] Becke A. D. (1993). J. Chem. Phys..

[cit29] Gordon M. S., Binkley J. S., Pople J. A., Pietro W. J., Hehre W. J. (1982). J. Am. Chem. Soc..

[cit30] Ditchfield R., Hehre W. J., Pople J. A. (1971). J. Chem. Phys..

[cit31] Miertuš S., Scrocco E., Tomasi J. (1981). Chem. Phys..

[cit32] Lenoid S. , Chemissian v4.67, http://www.chemissian.com/

[cit33] Gas K., Sawicki M. (2022). Materials.

[cit34] Sawicki M., Stefanowicz W., Ney A. (2011). Semicond. Sci. Technol..

[cit35] Daina A., Michielin O., Zoete V. (2017). Sci. Rep..

[cit36] Daina A., Zoete V. (2016). ChemMedChem.

[cit37] Banerjee P., Eckert A. O., Schrey A. K., Preissner R. (2018). Nucleic Acids Res..

[cit38] Lagunin A. A., Dubovskaja V. I., Rudik A. V., Pogodin P. V., Druzhilovskiy D. S., Gloriozova T. A., Filimonov D. A., Sastry N. G., Poroikov V. V. (2018). PLOS ONE.

[cit39] Quest Graph™ IC_50_ Calculator, AAT Bioquest, Inc., 2024, https://www.aatbio.com/tools/ic50-calculator

[cit40] Olive P. L., Banáth J. P. (2006). Nat. Protoc..

[cit41] Bernstein J., Davis R. E., Shimoni L., Chang N.-L. (1995). Angew. Chem., Int. Ed..

[cit42] Jelsch C., Ejsmont K., Huder L. (2014). IUCrJ.

[cit43] Tan S. S., Kim S. J., Kool E. T. (2011). J. Am. Chem. Soc..

[cit44] Ali M. A., Mirza A. H., Mei C. C., Bernhardt P. V., Karim M. R. (2013). Polyhedron.

[cit45] Akbar Ali M., Livingstone S. E. (1974). Coord. Chem. Rev..

[cit46] Bain G. A., Berry J. F. (2008). J. Chem. Educ..

[cit47] Lipinski C. A., Lombardo F., Dominy B. W., Feeney P. J. (2001). Adv. Drug Deliv. Rev..

[cit48] Ghose A. K., Viswanadhan V. N., Wendoloski J. J. (1999). J. Comb. Chem..

[cit49] Egan W. J., Merz K. M., Baldwin J. J. (2000). J. Med. Chem..

[cit50] Veber D. F., Johnson S. R., Cheng H.-Y., Smith B. R., Ward K. W., Kopple K. D. (2002). J. Med. Chem..

[cit51] Muegge I., Heald S. L., Brittelli D. (2001). J. Med. Chem..

[cit52] van Engeland M., Nieland L. J. W., Ramaekers F. C. S., Schutte B., Reutelingsperger C. P. M. (1998). Cytometry.

[cit53] Peña Q., Sciortino G., Maréchal J.-D., Bertaina S., Simaan A. J., Lorenzo J., Capdevila M., Bayón P., Iranzo O., Palacios Ò. (2021). Inorg. Chem..

