# Supplementary material for: Physicochemical properties and mechanism of action of a new copper(ii) pyrazine-based complex with high anticancer activity and selectivity towards cancer cells
Source: RSC Adv. 2024 Nov 12;14(49):36295–307. doi: 10.1039/d4ra06874b (PMC11556459; doi:10.1039/d4ra06874b)
Supplement: RA-014-D4RA06874B-s001 [file RA-014-D4RA06874B-s001.pdf]

Supplementary Information (SI) for RSC Advances.
This journal is © The Royal Society of Chemistry 2024

Parts of Figure S20 were not present in the original version. This has been updated here.

Supplementary Materials

Physicochemical properties and mechanism of action of the new copper(II) pyrazine-based complex with high anticancer activity and selectivity towards cancer cells

Table S1. SC-XRD experimental details for **Cu(L)** compound.

Chemical formula	$2(C_{14}H_{15}Cl_2CuN_5S_2)$
M_r	903.74
Crystal system, space group	Orthorhombic, $Pna2_1$
Temperature (K)	101
a, b, c (Å)	14.7703 (5), 9.2488 (3), 26.0606 (8)
V (Å^3)	3560.06 (19)
Z	4
Radiation type	Mo $K\alpha$
μ (mm^{-1})	1.77
Crystal size (mm)	0.59 × 0.22 × 0.02
No. of measured, independent and observed [$I > 2\sigma(I)$] reflections	28753, 9961, 8915
R_{int}	0.057
$(\sin \theta/\lambda)_{max}$ (Å^{-1})	0.748
$R[F^2 > 2\sigma(F^2)]$, $wR(F^2)$, S	0.048, 0.103, 1.04
No. of reflections	9961
No. of parameters	436
No. of restraints	1
Δ_{max}, Δ_{min} (e Å^{-3})	0.87, -0.91
Absolute structure	Refined as an inversion twin
Absolute structure parameter	0.504 (16)

Table S2. The sequences of primers used in the gene expression evaluation.

Gene symbol	Forward Sequence (5′→3′)	Reverse Sequence (5′→3′)
CAT	CCGGGACTACACCCAGATGA	TCTTGGCGTTCTCCTGATGC
GPX	CTTCAGGGTGGTATGGCTGT	TGGCCAGACCTTAATGTTCC
GSR	TCAGCTCACCACAACCTCTG	GAGACCAGCCTGACCAACAT
SOD2	CTTCAGGGTGGTATGGCTGT	TGGCCAGACCTTAATGTTCC
NF2L2	AGCTTAGCGTTCATCCGTGT	TCCAATCATCCGTCAAAACA
ATM	GCCGCGGTTGATACTACTTTG	GCAGCAGGGTGACAATAAACA
ATR	AATGGTTGGAGAATGCTGGC	ACATCACCCTTGGACCAGAG
OGG1	CCTGTGGGGACCTTATGCTG	TGTGAATCCCCTCTCCCGAT
PARP	CCCCACGACTTTGGGATGAA	AGACTGTAGGCCACCTCGAT
RNA18SN5	GAAACTGCGAATGGCTCATTAAA	CACAGTTATCCAAGTGGGAGAGG

Parts of Figure S20 were not present in the original version. This has been updated here.

ACTB	AGAGCTACGAGCTGCCTGAC	AGCACTGTGTTGGCGTACAG

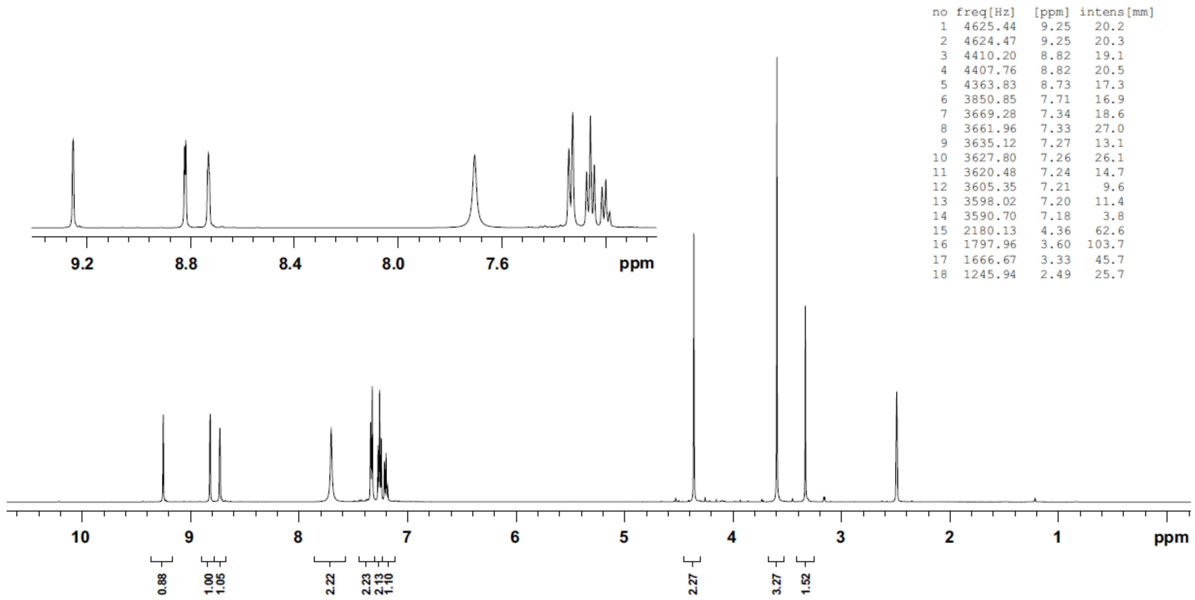

Figure S1. ^{1}H NMR spectrum of the **L** ligand.

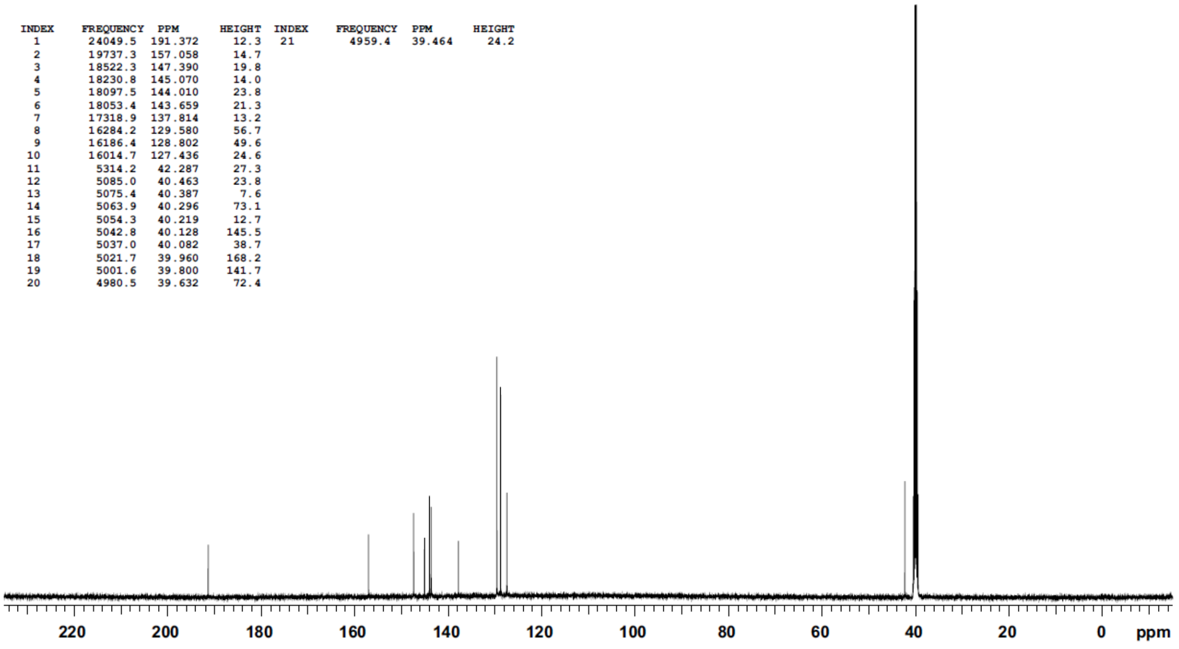

Figure S2. ^{13}C NMR spectrum of the **L** ligand.

Parts of Figure S20 were not present in the original version. This has been updated here.

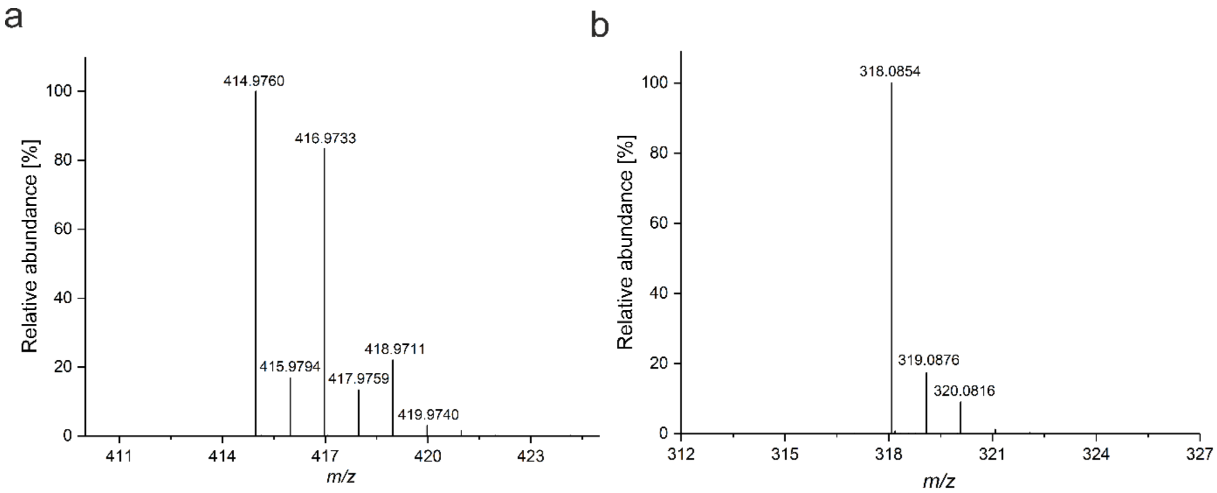

Figure S3. HR-ESI mass spectra obtained for the **Cu(L)** complex (a) and the ligand **L** (b). The experimental *m/z* value at 414.9760 (a) corresponds to $[M-Cl]^+$ (calculated *m/z* for $C_{14}H_{15}N_5S_2CuCl$: 414.9753). The experimental *m/z* value at 318.0854 (b) corresponds to $[M+H]^+$ (calculated *m/z* for $C_{14}H_{16}N_5S_2$: 318.0847).

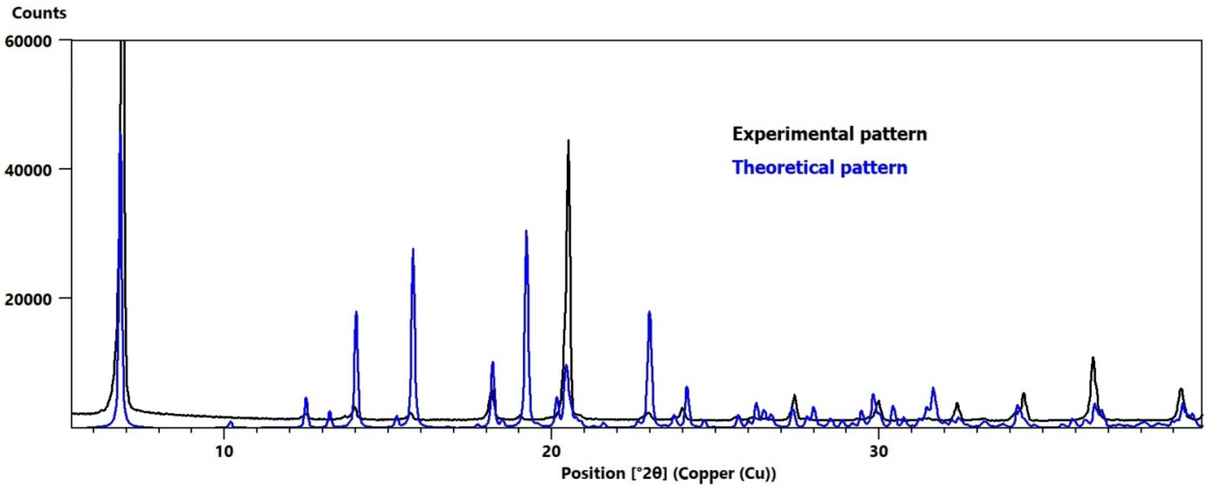

Figure S4. Theoretical and experimental PXRD patterns of the **Cu(L)** compound.

Parts of Figure S20 were not present in the original version. This has been updated here.

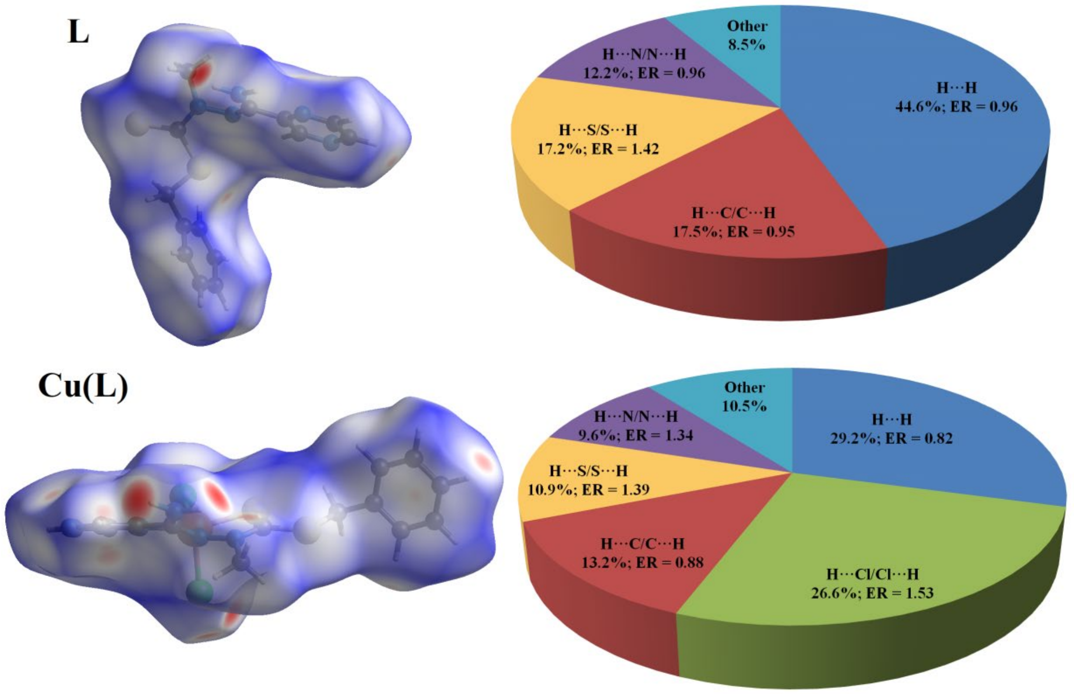

Figure S5. Hirschfeld Surfaces (left) mapped over d_{norm} and pie charts representing intermolecular contacts (right) predominant in the studied compounds, **L** and **Cu(L)** with their percentage involvement and Enrichment Ratios.

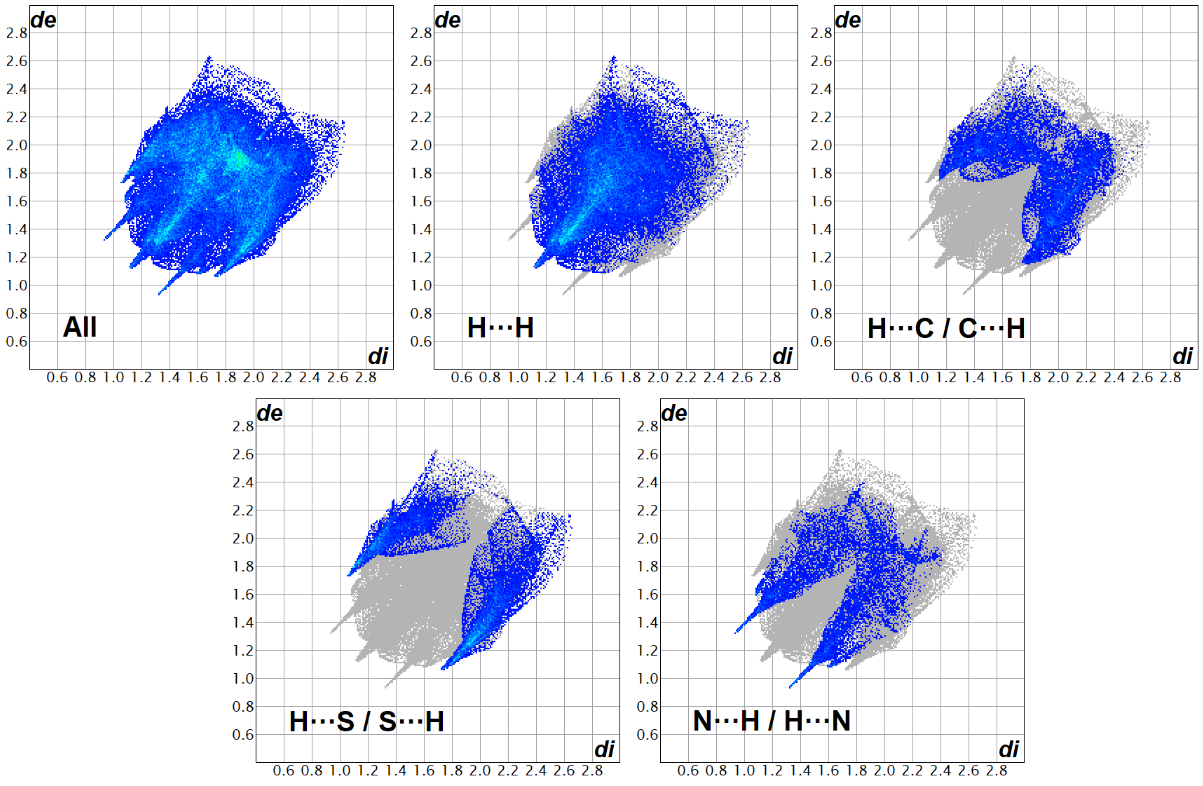

Figure S6. 2D Fingerprint Plots and the predominant contacts in **L** compound. *de* and *di* are the distances to the nearest atomic exterior and interior to the surface.

Parts of Figure S20 were not present in the original version. This has been updated here.

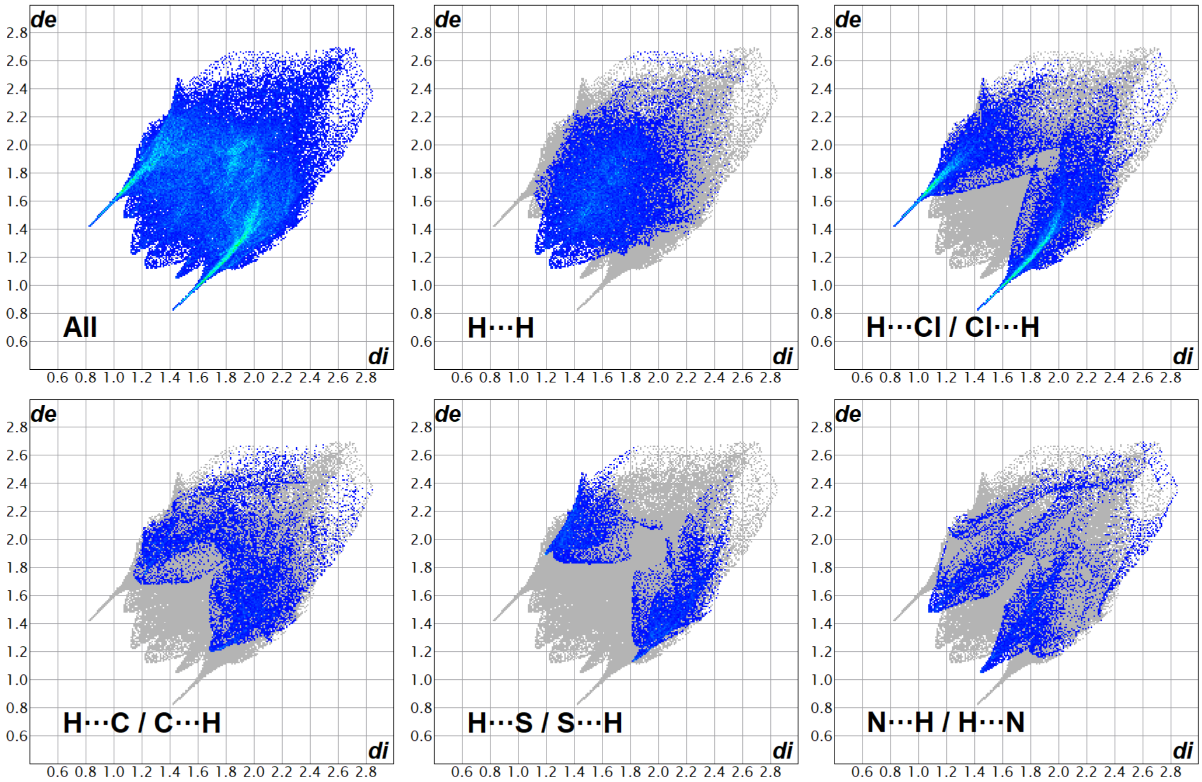

Figure S7. 2D Fingerprint Plots and the predominant contacts in **Cu(L)** compound. *de* and *di* are the distances to the nearest atomic exterior and interior to the surface.

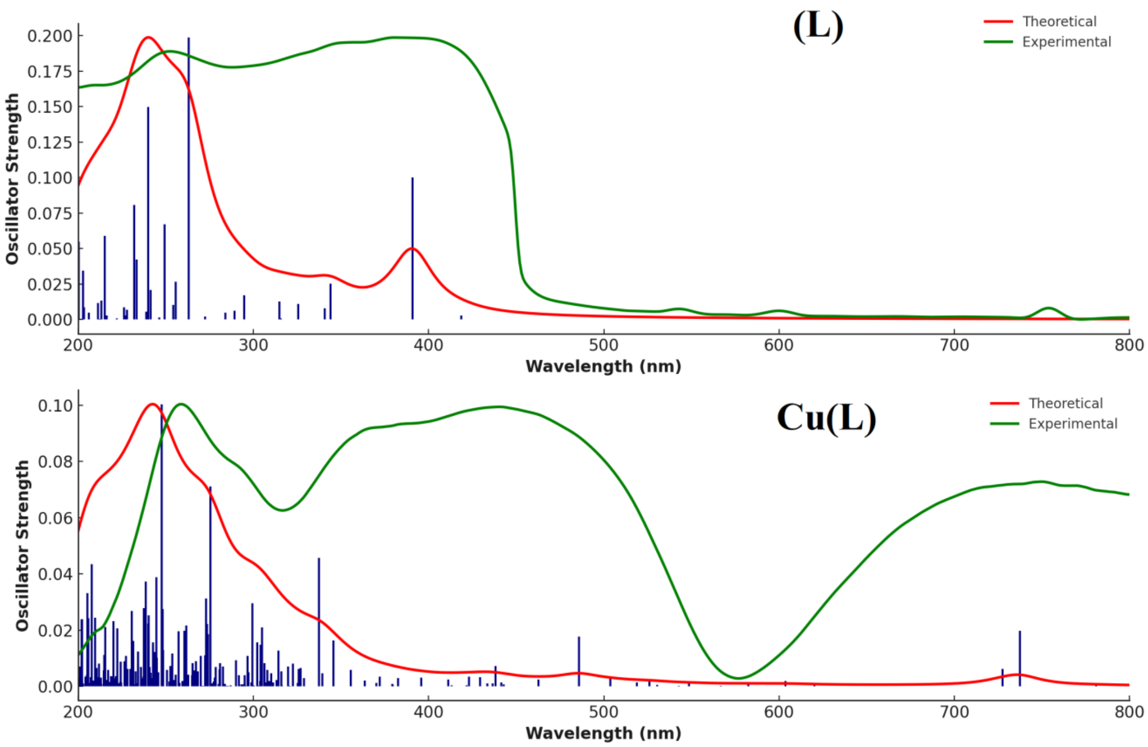

Figure S8. Experimental (green) and calculated (red) UV-Vis spectra of the studied compounds: **L** and **Cu(L)**. Oscillator strengths are represented as vertical navy-blue lines.

Table S3. The most important electronic transitions appearing in the studied compounds and the fluorescence emission maximum of **L** and **Cu(L).**

Parts of Figure S20 were not present in the original version. This has been updated here.

Absorption spectroscopy					
X-ray determined coordinates					
Experimental		Calculated			
**L** (nm)	**Cu(L)** (nm)	**L** (nm)	**Cu(L)** (nm)	*f*	Orbital transition
253	258	239.9		0.150	π→π*
			247.8	0.100	n/π →π*
		263.1		0.199	n/π →π*
	370		275.4	0.071	n/π/σ/d(Cu)→ π*/d(Cu)
			337.5	0.046	n/σ/d(Cu)→ π*/d(Cu)
382		390.7		0.100	n/π →π*
	442		485.7	0.018	n/σ/d(Cu)→π*/d(Cu)
	749		737.4	0.020	n/d(Cu)→π*/d(Cu)

DMSO solution			
Experimental	Calculated		
**Cu(L)** (nm)	**Cu(L)** (nm)	*f*	Orbital transition
216	218.1	0.097	n/d(Cu)→σ*
	243.9	0.081	π/d(Cu)→π*
278	322.6	0.029	n/d(Cu)→π*
340	350.1	0.035	π→d(Cu)/π*
540	468.4	0.024	n/d(Cu)→π*
701	605.4	0.012	n/d(Cu)→π*

Fluorescence			
Compound	ExW	EmW	Intensity
**L**	470	481	36
**Cu(L)**	-	-	-

Used abbreviations: *f* - oscillator strength, d(Cu) — d orbital of a copper cation, σ — σ orbital of the organic ligand, n — non-bonding orbital, π — π orbital of the organic ligand, * — antibonding orbital.

Parts of Figure S20 were not present in the original version. This has been updated here.

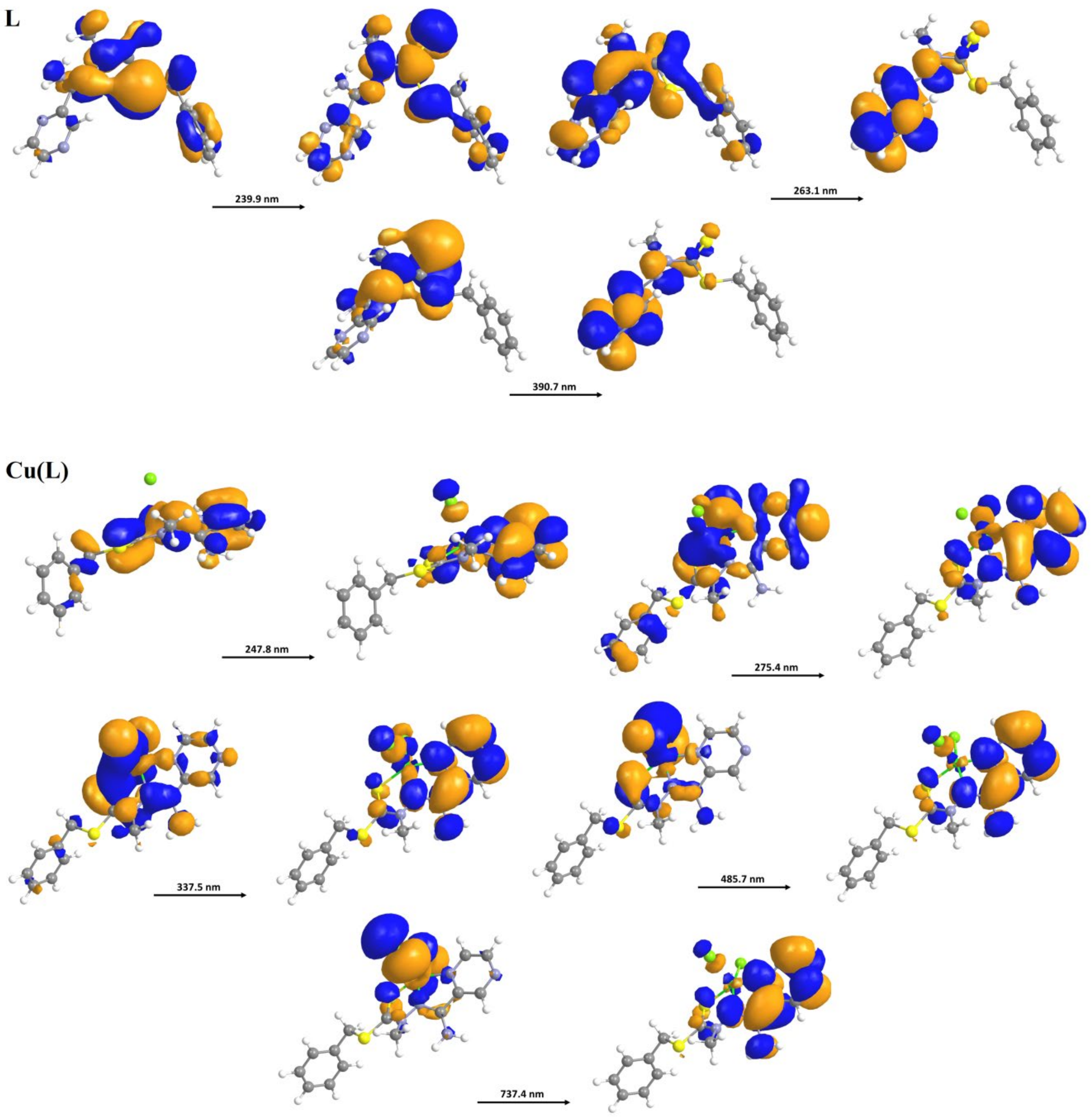

Figure S9. The main molecular orbital transitions in the studied compounds, **L** and **Cu(L)**, which are associated with the solid state absorption spectra of these compounds.

Parts of Figure S20 were not present in the original version. This has been updated here.

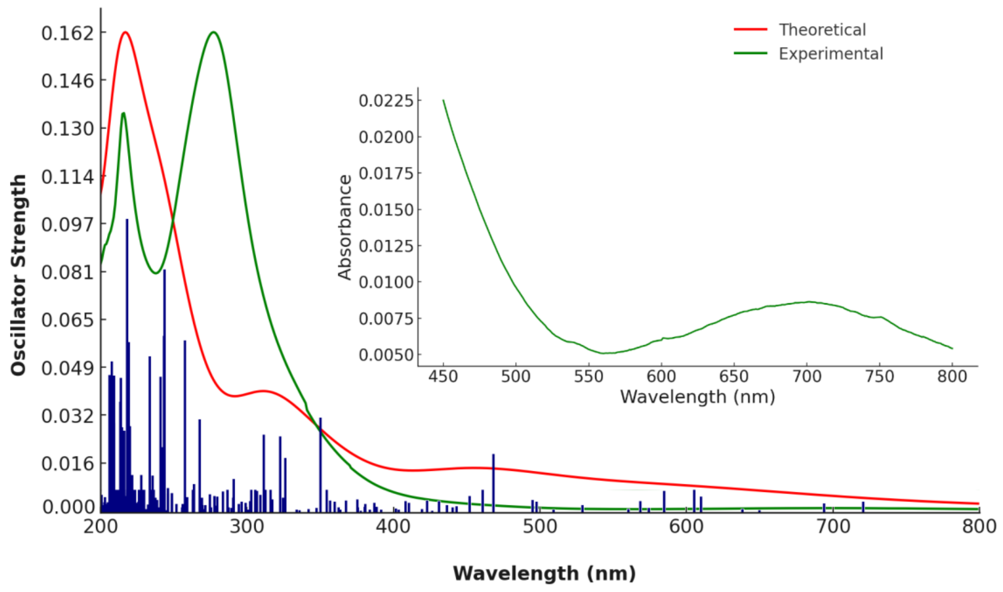

Figure S10. Experimental (green) and calculated (red) UV-Vis spectra of **Cu(L)** in DMSO. Oscillator strengths are represented as vertical navy-blue lines.

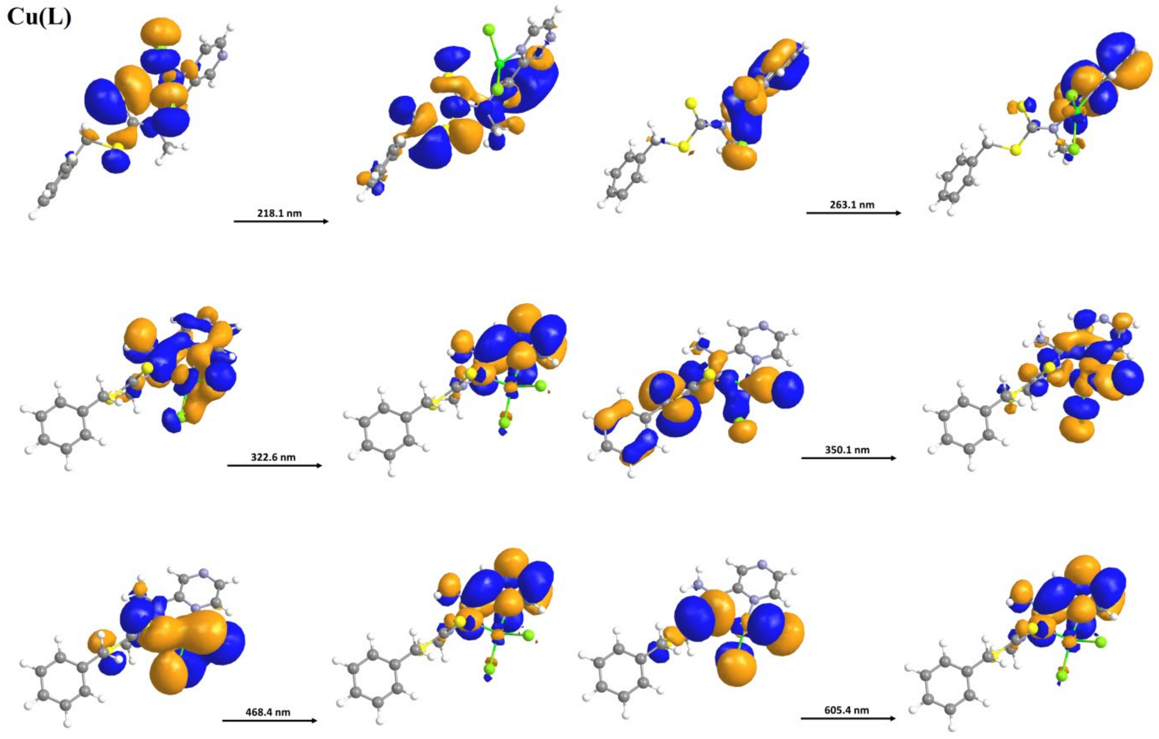

Figure S11. The main molecular orbital transitions for **Cu(L)** in DMSO, which are associated with the solution absorption spectra of this compound.

Parts of Figure S20 were not present in the original version. This has been updated here.

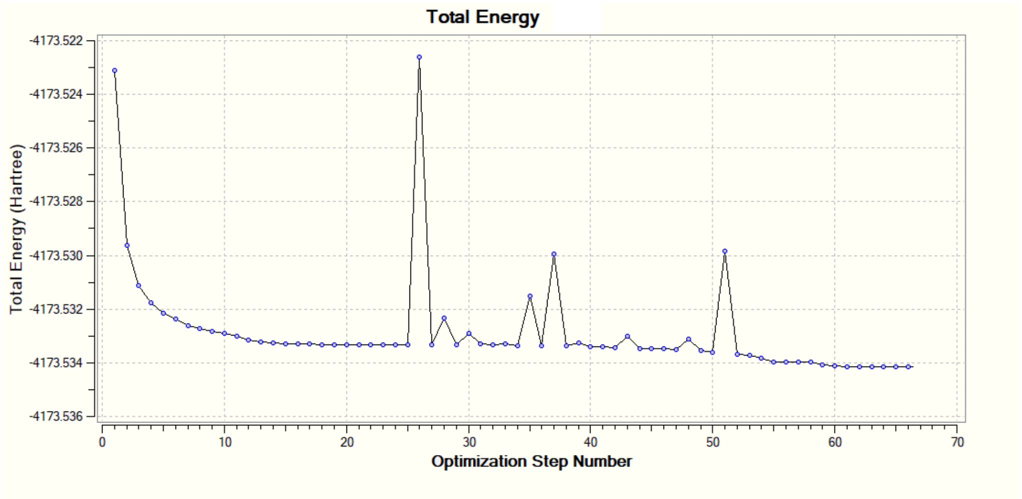

Figure S12. Changes in total energy during optimization of **Cu(L)** complex in water, showing overall decrease in energy, suggesting the system's energetic stability in aqueous environment.

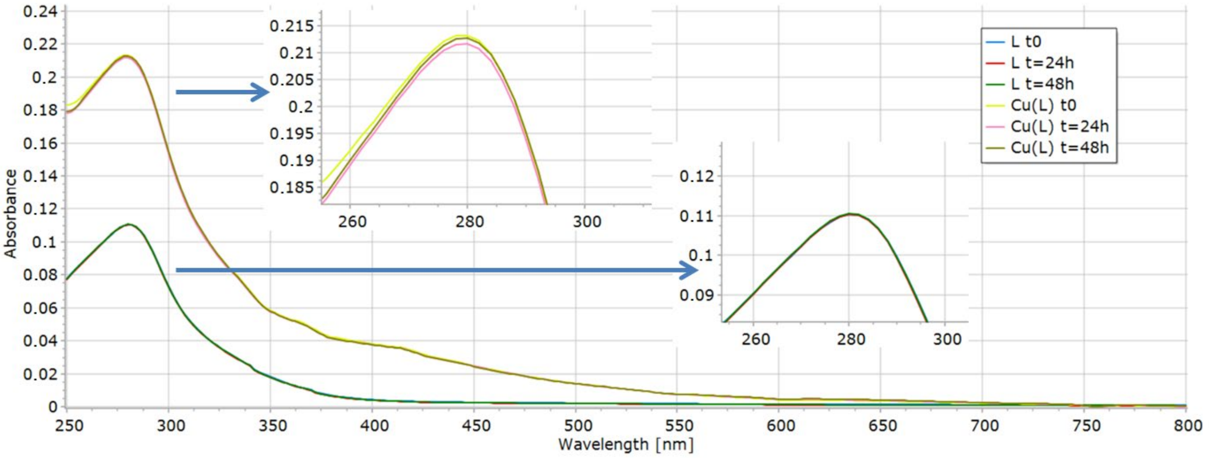

Figure S13. Time-dependent UV-Vis-monitored stability of **L** and **Cu(L)** in DMSO/H_2O (1:1) mixture (C = 5 μM; T = 24°C).

Parts of Figure S20 were not present in the original version. This has been updated here.

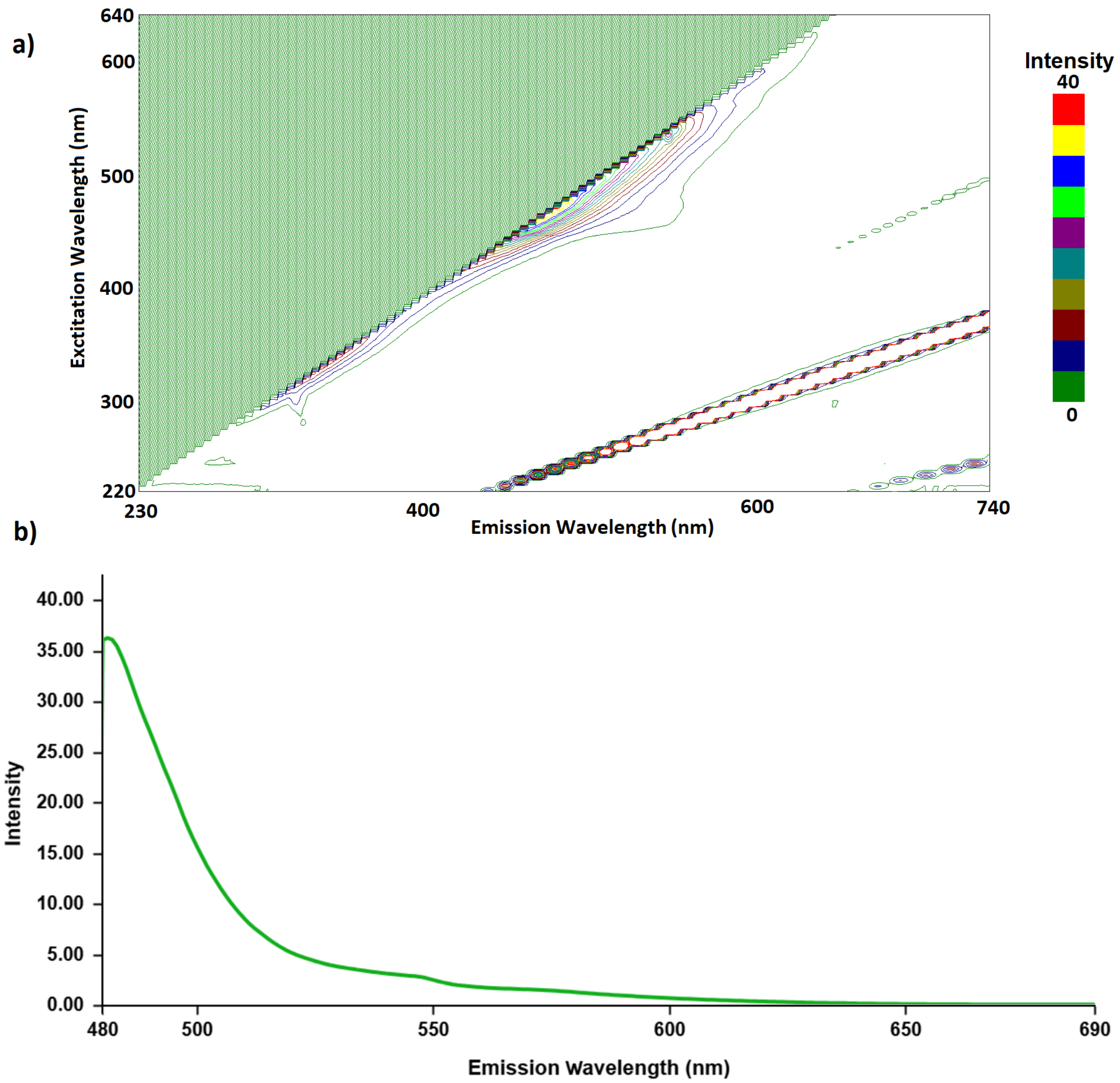

Figure S14. 3D fluorescence spectrum of ligand **L** (a) and 2D fluorescence spectrum of ligand **L** (b) obtained as a cross-section of the 3D spectrum at an excitation wavelength of 470 nm.

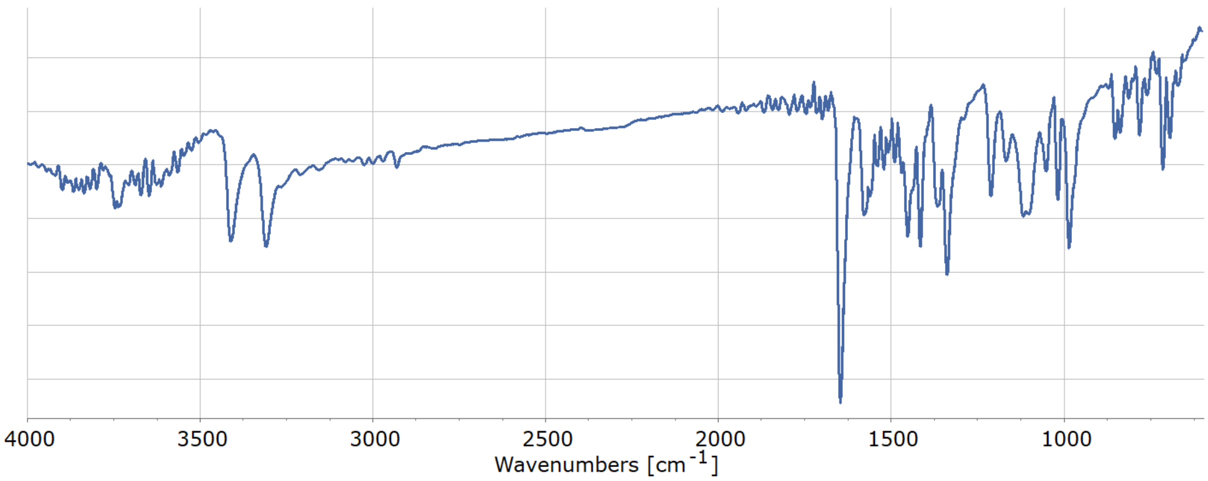

Figure S15. Experimental FTIR spectrum of the **L** ligand.

Parts of Figure S20 were not present in the original version. This has been updated here.

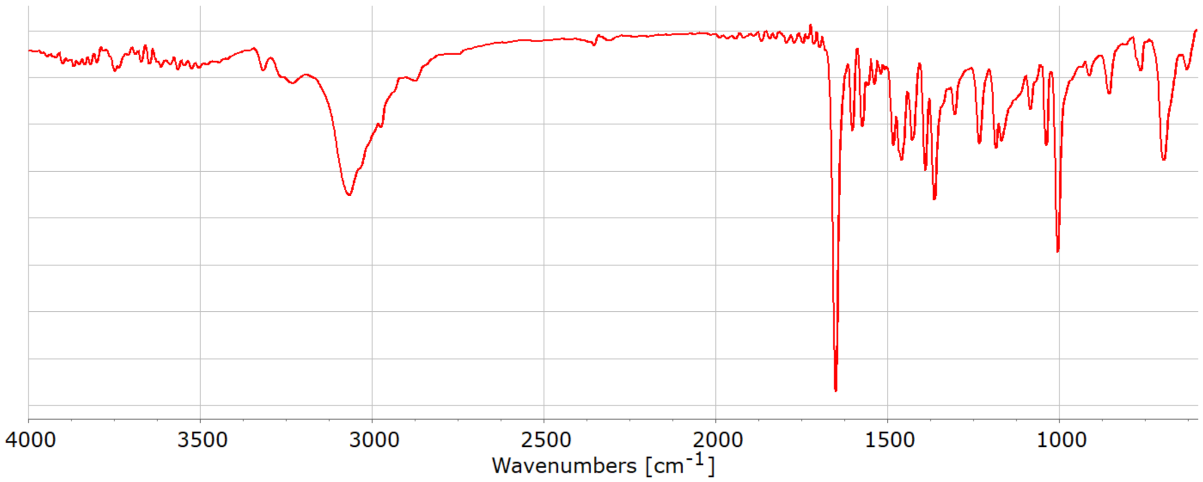

Figure S16. Experimental FTIR spectrum of the **Cu(L)** compound.

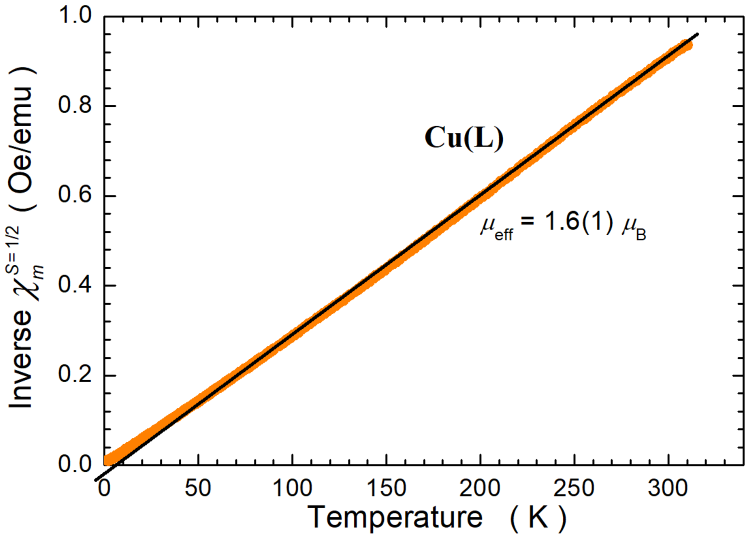

Figure S17. Temperature dependence of the inverse of the molar *spin* susceptibility of Cu ions, $\chi_m^{S=1/2}$ in the **Cu(L)** coordination compound (orange symbols). The straight black line approximates 1/T paramagnetic dependency. Its slope yields the magnitude of the inverse of the Curie constant, from which the value of the effective spin moment μ_{eff} is calculated and given in the units of Bohr magnetons μ_B.

Parts of Figure S20 were not present in the original version. This has been updated here.

Pa	Pi	Cell-line	Description	Tissue/Organ	Type	IAP
0.633	0.004	A-375	Malignant melanoma	Skin	Melanoma	0.931
0.591	0.025	HeLa	Cervical adenocarcinoma	Cervix	Adenocarcinoma	0.885
0.558	0.004	NCI-H1299	Non-small cell lung carcinoma	Lung	Carcinoma	0.916
0.430	0.050	YAPC	Pancreatic carcinoma	Pancreas	Carcinoma	0.817
0.378	0.132	HCC1937	Breast Carcinoma	Breast	Carcinoma	0.806
0.363	0.104	SNU-5	Gastric Carcinoma	Stomach	Carcinoma	0.828
0.360	0.076	HGC-27	Gastric carcinoma	Stomach	Carcinoma	0.830
0.339	0.066	DU-145	Prostate carcinoma	Prostate	Carcinoma	0.896
0.339	0.122	HT1197	Carcinoma	Urinary bladder	Carcinoma	0.815
0.330	0.236	SK-LU-1	Adenocarcinoma	Lung	Carcinoma	0.810
0.328	0.050	HT	Lymphoma	Haematopoietic and lymphoid tissue	Leukemia	0.857
0.318	0.191	NCI-H358	Bronchioalveolar Carcinoma	Lung; Bronchiole	Carcinoma	0.805
0.314	0.136	KYSE-520	Esophageal squamous cell carcinoma	Esophagus	Carcinoma	0.858
0.314	0.272	GIST882	Gastrointestinal stromal tumor	Intestine	Carcinoma	0.824
0.305	0.240	NCI-H441	Papillary adenocarcinoma	Lung	Adenocarcinoma	0.813

Figure S18. Cancer cell lines the compound **L** is predicted to be active against. Pa – probably active; Pi – probably inactive; IAP - Invariant Accuracy of Prediction. Only results with a Pa value higher than 0.300 are included.

Parts of Figure S20 were not present in the original version. This has been updated here.

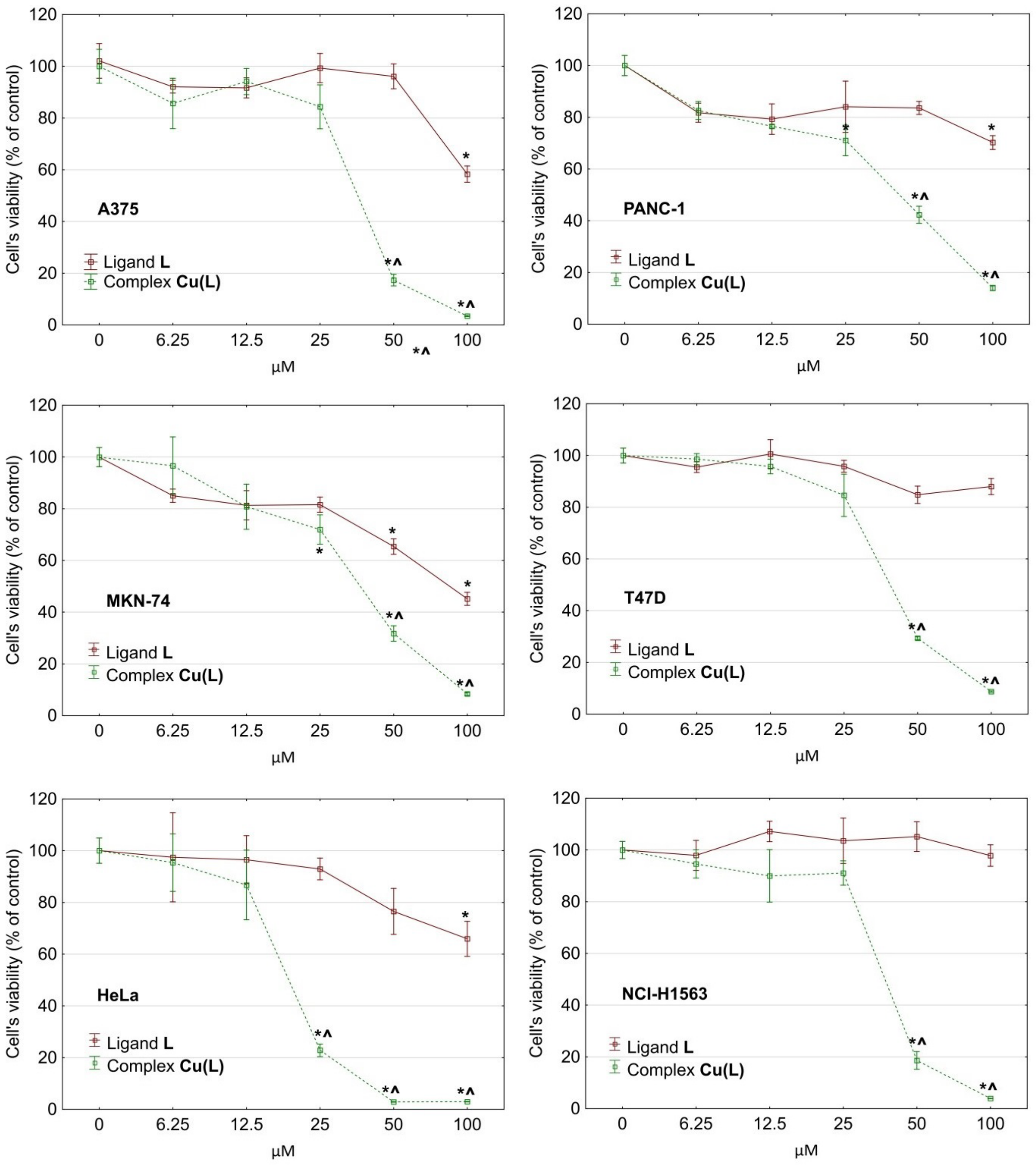

Figure S19. Cytotoxicity of ligand **L** and its complex, **Cu(L)**, based on MTT test results after 48 h incubation. Data are presented as % of control culture viability (mean±SD). *$p<0.05$ vs. control, ^ $p<0.05$ vs. ligand **L**.

Parts of Figure S20 were not present in the original version. This has been updated here.

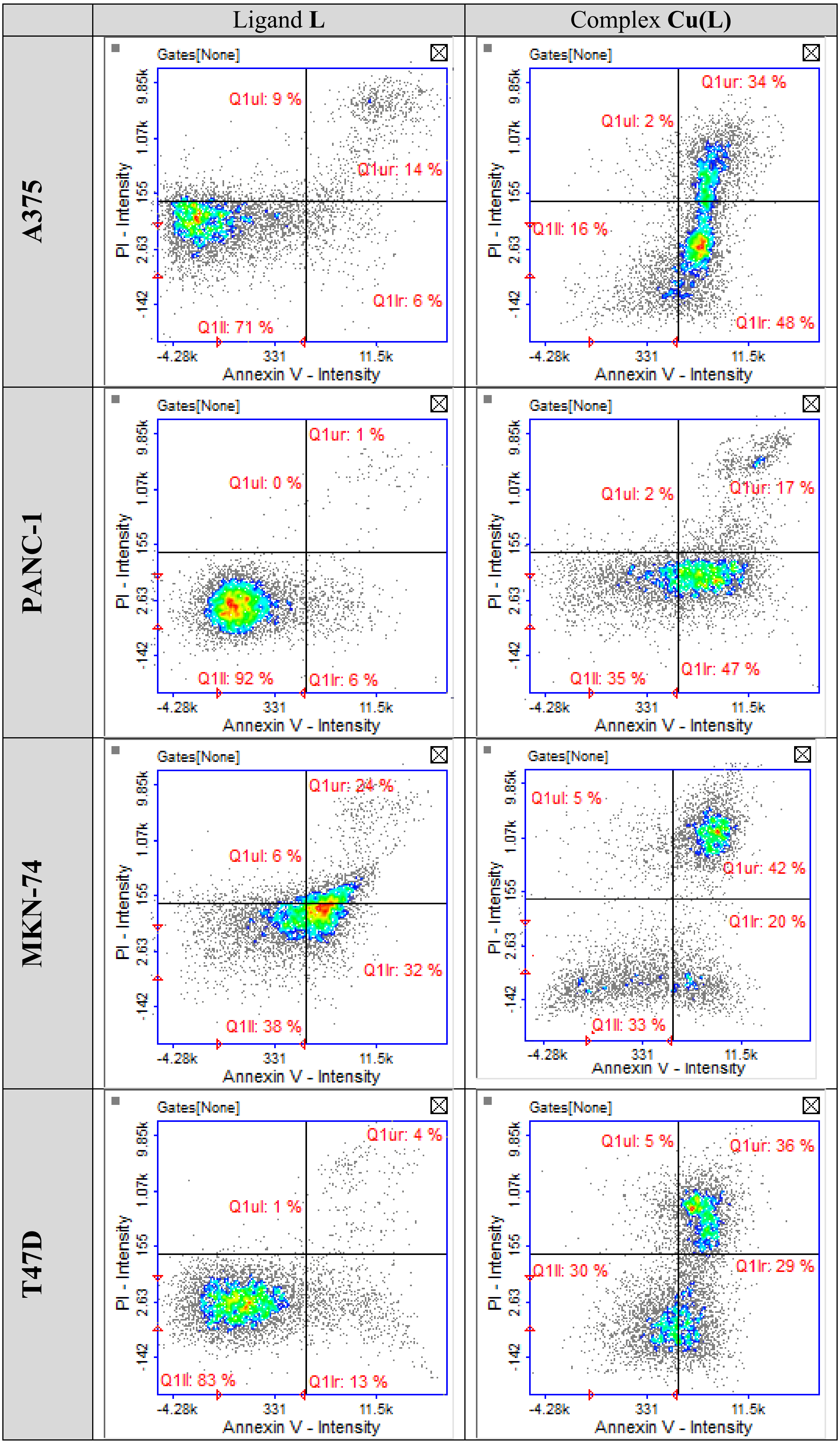

Parts of Figure S20 were not present in the original version. This has been updated here.

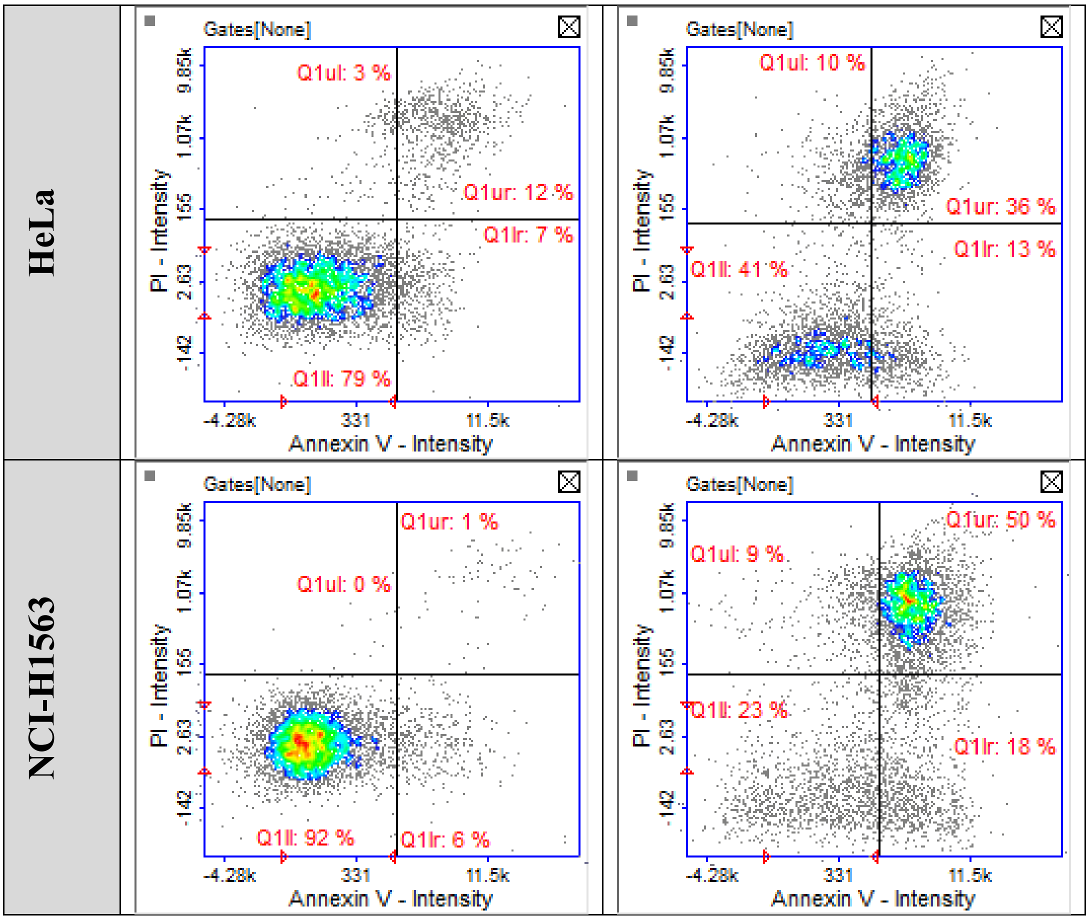

Figure S20. Apoptosis/necrosis detection by image cytometry. The results show one representative experiment. Q1II—live, Q1Ir—early apoptotic, Q1ur—late apoptotic, and Q1uI—necrotic cells.

Parts of Figure S20 were not present in the original version. This has been updated here.

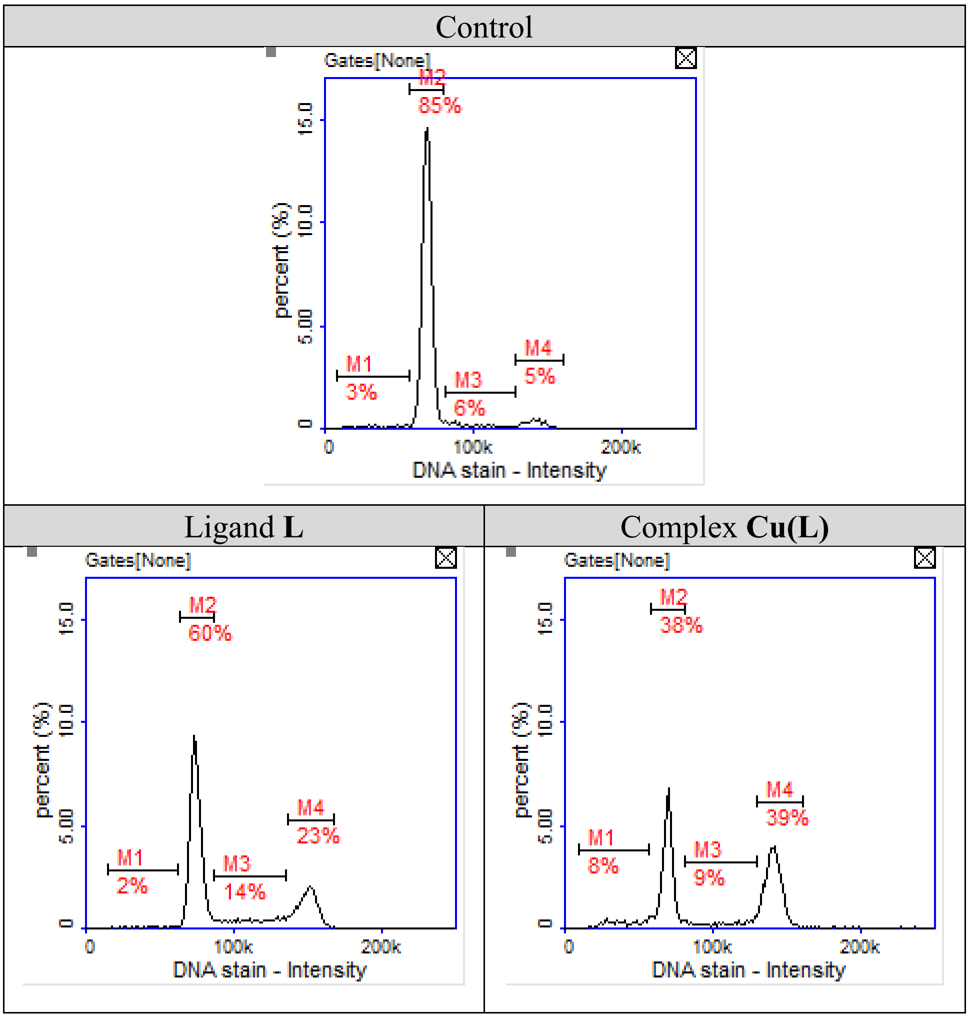

Figure S21. Representative histograms of cell cycle analysis in HeLa cells treated with ligand **L** (100 µM) and its complex, **Cu(L)** (in concentration corresponding to IC_{50} value – 17.50 µM) or DMSO as a vehicle in control culture for 48 h. M1—subG1, M2—G1, M3—S, M4—G2/M phase.

Parts of Figure S20 were not present in the original version. This has been updated here.

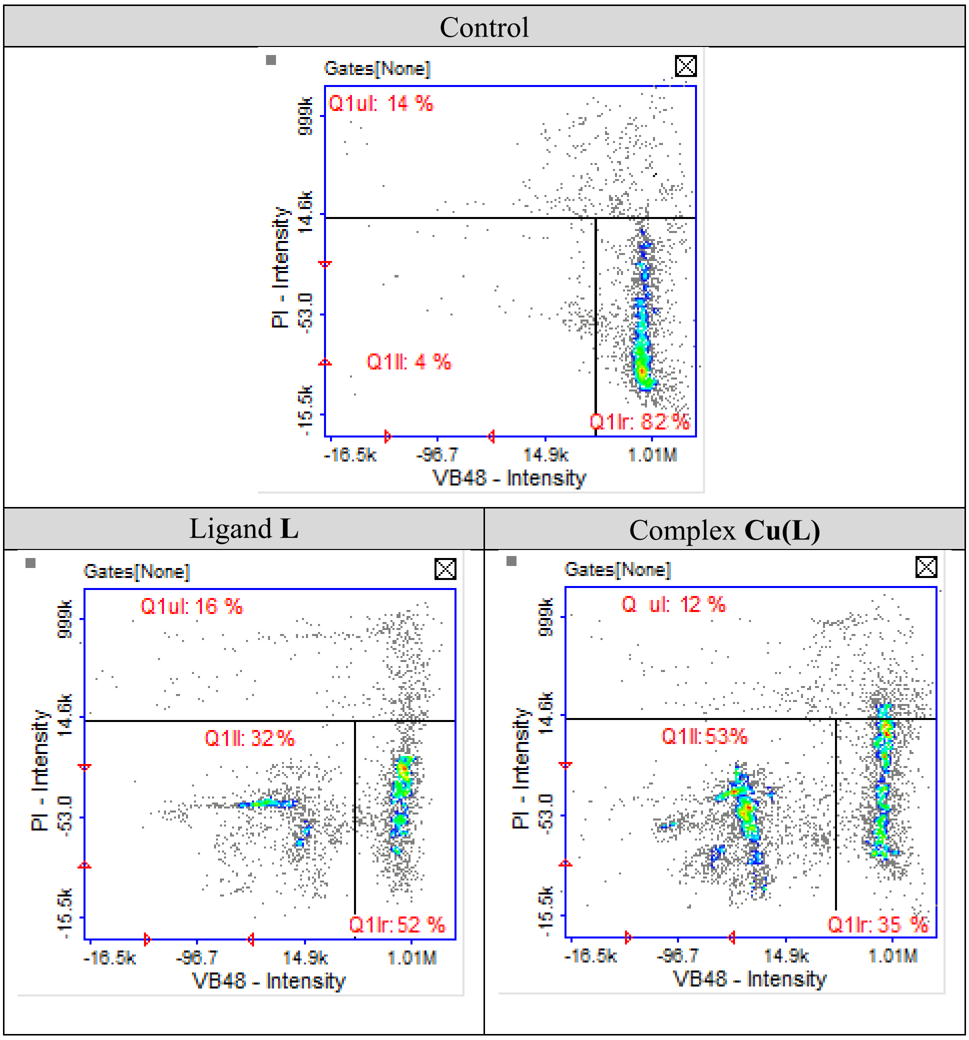

Figure S22. Representative histograms of thiol level distribution in HeLa cells treated with ligand **L** (100 µM) and its complex, **Cu(L)** (in concentrations corresponding to IC_{50} value – 17.50 µM) or DMSO as a vehicle in control culture for 48 h. Cells were stained with VitaBright-48™ and propidium iodide (PI). Q1lr – healthy cells, Q1ll – subpopulation with decreased thiol levels, Q1ul – dead cells (PI-positive).

Parts of Figure S20 were not present in the original version. This has been updated here.

Table S4. Antimicrobial activity of tested compounds presented as minimal inhibitory concentration (MIC), minimal bactericidal concentration (MBC), and minimal fungicidal concentration (MFC) [mg/L].

Microorganism	**Compound**				**Reference**
	Ligand **L**		Complex **Cu(L)**		Vancomycin
**Gram-positive bacteria**	**MIC**	**MBC**	**MIC**	**MBC**	**(Van)**
S. aureus ATCC 25923	>500	>500	250	500	0.98
S. aureus ATCC BAA-1707*	>500	>500	250	250	0.98
S. epidermidis ATCC 12228	>500	>500	250	500	0.98
M. luteus ATCC 10240	500	>500	250	250	0.12
E. faecalis ATCC 29212	>500	>500	250	>500	1.95
B. cereus ATCC 10876	>500	>500	250	250	0.98
**Gram-negative bacteria**	**MIC**	**MBC**	**MIC**	**MBC**	**(Cip)**
S. typhimurium ATCC 14028	>500	>500	>500	>500	0.061
E. coli ATCC 25922	>500	>500	>500	>500	0.015
P. mirabilis ATCC 12453	>500	>500	>500	>500	0.03
K. pneumoniae ATCC 13883	>500	>500	>500	>500	0.12
P. aeruginosa ATCC 9027	>500	>500	>500	>500	0.49
**Yeasts**	**MIC**	**MFC**	**MIC**	**MFC**	**(Nys)**
C. albicans ATCC 102231	>500	>500	250	>500	0.24
C. parapsilosis ATCC 22019	>500	>500	62.5	>500	0.24
C. glabrata ATCC 90030	>500	>500	>500	>500	0.48

* Methicillin-resistant *Staphylococcus aureus* (MRSA)
